# Small Molecular Inhibitors Reverse Cancer Metastasis by Blockading Oncogenic PITPNM3

**DOI:** 10.1002/advs.202204649

**Published:** 2022-10-26

**Authors:** Zihao Liu, Yu Shi, Li Lv, Jianing Chen, WenG. Jiang, Jun Li, Qun Lin, Xiaolin Fang, Jingbo Gao, Yujie Liu, Qiang Liu, Xiaoding Xu, Erwei Song, Chang Gong

**Affiliations:** ^1^ Breast Tumor Center Sun Yat‐sen Memorial Hospital Sun Yat‐sen University Guangzhou 510120 P. R. China; ^2^ Guangdong Provincial Key Laboratory of Malignant Tumor Epigenetics and Gene Regulation Sun Yat‐sen Memorial Hospital Sun Yat‐sen University Guangzhou 510120 P. R. China; ^3^ Cardiff China Medical Research Collaborative School of Medicine Cardiff University Heath Park Cardiff CF14 4XN UK; ^4^ Department of Biochemistry Zhongshan School of Medicine Sun Yat‐sen University Guangzhou 510080 P. R. China; ^5^ Department of Pharmacy Sun Yat‐Sen Memorial Hospital Sun Yat‐Sen University Guangzhou 510120 P. R. China; ^6^ Department of Breast and Thyroid Surgery Shenzhen People's Hospital The Second Clinical Medical College of Jinan University The First Affiliated Hospital of Southern University of Science and Technology Shenzhen 518020 P. R. China

**Keywords:** metastasis, pan‐cancer analysis, PITPNM3, small molecular inhibitors

## Abstract

Most cancer‐related deaths are a result of metastasis. The development of small molecular inhibitors reversing cancer metastasis represents a promising therapeutic opportunity for cancer patients. This pan‐cancer analysis identifies oncogenic roles of membrane‐associated phosphatidylinositol transfer protein 3 (PITPNM3), which is crucial for cancer metastasis. Small molecules targeting PITPNM3 must be explored further. Here, PITPNM3‐selective small molecular inhibitors are reported. These compounds exhibit target‐specific inhibition of PITPNM3 signaling, thereby reducing metastasis of breast cancer cells. Besides, by using nanoparticle‐based delivery systems, these PITPNM3‐selective compounds loaded nanoparticles significantly repress metastasis of breast cancer in mouse xenograft models and organoid models. Notably, the results establish an important metastatic‐promoting role for PITPNM3 and offer PITPNM3 inhibition as a therapeutic strategy in metastatic breast cancer.

## Graphical abstract

Pan‐cancer analysis identifies oncogenic roles of membrane‐associated phosphatidylinositol transfer protein 3 (PITPNM3). Small molecule inhibitors targeting PITPNM3 exhibit target‐specific inhibition of PITPNM3 signaling, thereby reducing metastasis of breast cancer. By using nanoparticle‐based delivery systems, PITPNM3‐selective compounds loaded nanoparticles significantly repress metastasis of breast cancer in vivo and in vitro.

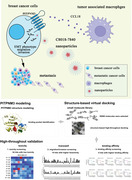

## 1 Introduction

Despite the significant progress that had been made over chemotherapy agents, immunotherapy, and targeted therapy agents, most of cancer patients still suffer metastatic disease. Cancer metastatic disease account for the most majority of cancer related mortality. In certain cancers, even if diagnostic advances and intense treatment of cancer, cancer patients with metastatic disease would have poor prognosis whose survival time is measured in months. Thus it is urgent to develop novel therapeutic agents for preventing and treating metastasis of cancer patients.

Tumor microenvironment (TME), which comprises malignant tumor cells, tumor associated macrophages (TAMs), tumor associate fibroblast, and other immune components such as: dendritic cells, T cells, and a variety of surrounding stromal cells, plays an important role in cancer metastasis.^[^ ^1^ ^]^ In TME of cancers, TAMs are one of the most prominent components of TME and can promote distant metastasis, innate immunity, as well as adaptive immunity by secreting several cytokines and chemokines.^[^ ^2^ ^]^ It was first reported that membrane‐associated phosphatidylinositol transfer protein 3 (PITPNM3) acts as a functional binding receptor for C‐C motif chemokine ligand (CCL18), which is abundantly produces by TAM.^[^ ^3^ ^]^ Upon binding, PITPNM3 phosphorylates its downstream factor Protein Tyrosine Kinase 2 beta (PTK2B, known as PYK2) to promote epithelial‐mesenchymal transition (EMT), migration, invasion, and metastasis of malignant cancer cell. Cancer metastasis driven by PITPNM3 has been reported in breast cancer (BRCA),^[^ ^3^, ^4^ ^]^ hepatocellular carcinoma,^[^ ^5^ ^]^ and pancreatic ductal carcinoma.^[^ ^6^ ^]^ However, oncogenic role of PITPNM3 is undetermined in pan‐cancer. In this study we performed systematic analysis of PITPNM3 in pan‐cancer and revealed oncogenic role of PITPNM3. Therefore, PITPNM3 is a potential target for tumor metastasis.

PITPNMs (PITPNM1, PITPNM2, and PITPNM3) is human homologues of Drosophila retinal degeneration B (rdgB) proteins.^[^ ^7^ ^]^ Unlike its human homologs PITPNM1 and PITPNM2 which are highly enriched in endoplasmic reticula and Golgi complex, PITPNM3 is a membrane‐bound receptor.^[^ ^3^, ^7^ ^]^ PITPNM3 lacks an N‐terminal phospholipids transfer domain but contains a Ca^+2^ binding domain, transmembrane domains, and a PTK2B binding domain.^[^ ^7^, ^8^ ^]^ PITPNM3 interacts with the four‐point‐one, ezrin, radixin, moesin (FERM) domain of PTK2B, and phosphorylates PTK2B through its PTK2B‐binding domain.^[^ ^7^, ^9^ ^]^ Considering that therapeutic approaches targeting PITPNM3 are still lacking, we executed compound screening to selectively identify small molecule inhibitors targeting PITPNM3 for the treatment of breast cancer metastasis.

## 2 Results

### 2.1 Pan‐Cancer Landscape Identified Oncogenic Role of PITPNM3

**Figure 1:**
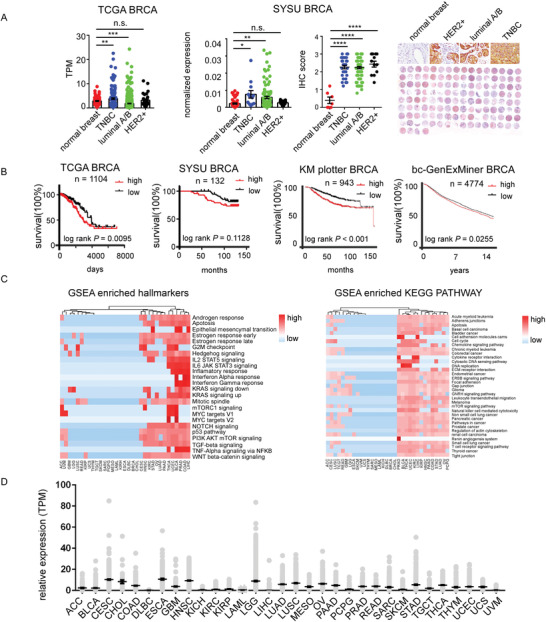
An comprehensive pan‐cancer analysis revealed oncogenic role of PITPNM3. A) The expression of PITPNM3 in The Cancer Genome Atlas (TCGA) breast cancer (BRCA) and Sun Yat‐Sen University (SYSU) cohort BRCA. B) The prognosis roles of PITPNM3 in TCGA BRCA, SYSU BRCA, KM plotter BRCA, and bc‐GenExMiner BRCA. C) GSEA hallmarkers analysis and GSEA KEGG pathway analysis in pan‐cancer. D) The expression of PITPNM3 in different types of cancer from TCGA datasets. *p < 0.05, **p < 0.01, ***p < 0.001.

Previously, we have revealed that PITPNM3 can promote breast cancer (BRCA) cell EMT transformation, migration, and invasion. To confirm the oncogenic role of PITPNM3, we analyzed the prognostic roles of PITPNM3. In The Cancer Genome Atlas (TCGA) and Sun Yat‐Sen University (SYSU) cohort, the expression of PITPNM3 is much high in triple negative cancer than solid normal (**Figure** 1A,B). High expression of PITPNM3 significantly predicts poor prognosis of breast cancer in TCGA BRCA, KM plotter BRCA, bc‐GenExMiner BRCA and marginally predicts poor overall survival of breast cancer in SYSU cohort (Figure 1A,B). In the Genotype‐Tissue Expression (GTEx) datasets, PITPNM3 is much higher in the spleen, cerebellum, and ovary tissue (Figure S1A, Supporting Information). Since PITPNM3 can be activated by binding with C‐C Motif Chemokine Ligand 18 (CCL18), the relationship between CCL18, PITPNM3 and breast cancer prognosis was determined. The protein level of CCL18 was determined by immunohistochemistry (IHC) and the expression of CCL18 in TCGA pan‐cancer was extracted (Figure S1B,C, Supporting Information). The overall survival of PITPNM3^high^ CCL18^high^ breast cancer patients was significantly worse than PITPNM3^low^CCL18^low^ patients in the TCGA and SYSU cohort (Figure S1D, Supporting Information). These data suggested that a high level of PITPNM3 is associated with poor prognosis and targeting PITPNM3 might be promising for breast cancer patients. To determine whether patients carrying other types of malignancy could benefit from targeting PITPNM3 therapy, we performed a systematic analysis of PITPNM3 in pan‐cancer. Gene Set Enrichment Analysis (GSEA) hallmarkers analysis showed cancer samples with high expression of PITPNM3 could be enriched with metastasis hallmarkers such as: epithelial mesencymal transition, transforming growth factor‐beta (TGF‐beta) signaling and WNT‐beta‐catenin signaling; tumor progression hallmarkers such as: KRAS signaling, MYC target, PI3K signaling, G2M checkpoint and mitotic spindle; immune response hallmarkers such as: interleukin‐2 (IL2)—Signal transducer and activator of transcription (STAT5) signaling, IL6 STAT3 signaling and interferon (IFN) alpha/gamma signaling in most types of cancer (Figure 1C). To strengthen our analysis, we performed GSEA Kyoto Encyclopedia of Genes and Genomes (KEGG) analysis, consistently, high expression of PITPNM3 could enrich in metastasis KEGG pathways such as: adherens junctions, extracellular matrix (ECM) receptor interaction, focal adhesion, and regulation of actin skeleton; tumor associated KEGG pathways such as: acute myeloid leukemia, basal cell carcinoma, bladder cancer, colorectal cancer, and other types of cancer pathways; immune KEGG pathways such as: leukocyte transendothelial migration, natural killer cell mediated cytotoxicity, and T cell signaling in most types of cancer (Figure 1C). Survival analysis showed that high expression of PITPNM3 predicts poor survival of skin cutaneous melanoma (SKCM) and marginally predicts poor overall survival of kidney chromophobe renal cell carcinoma (KICH), ovarian serous cystadenocarcinoma (OV), pancreatic adenocarcinoma (PAAD), pheochromocytoma and paraganglioma (PCPG, Figure S2, Supporting Information), while high expression of CCL18 predicts poor survival of glioblastoma multiforme (GBM), OV, thymoma (THYM), and uterine carcinosarcoma (UCS, Figure S3, Supporting Information). The co‐expression of CCL18 and PITPNM3 predicts poor survival of GBM, KICH, PCPG, and UCS (Figure S4, Supporting Information). The expression of PITPNM3 is dynamics in different cancer (Figure 1D). To further confirm these results, the expression of PITPNM3 in Cancer Cell Line Encyclopedia (CCLE) was collected (Figure S5A, Supporting Information) and 13 types of cancer cell lines in which PITPNM3 are highly associated with their progression screened by GSEA and KEGG analysis (Figure 1C; Figure S5B, Supporting Information) were selected. Knockdown of PITPNM3 by siRNAs will significantly abrogate CCL18 conducted cell metastasis in these 13 cancer cell lines (Figure S5C, Supporting Information). These results indicate that PITPNM3 plays an oncogenic role in pan‐cancer and targeting PITPNM3 might be promising for cancer treatment. Early studies revealed that PITPNM3 interacted with PTK2B through its PTK2B binding domain and could phosphorylate PTK2B upon activation.^[^ ^3^, ^7^ ^]^ To confirm these results, we employed a reported clustered regularly interspaced short palindromic repeat/CRISPR‐associated nuclease 9 (CRISPR/Cas9) plasmid to insert a stop codon before PTK2B binding domain to acquire an endogenous PTK2B‐binding‐domain deletion MDA‐MB‐231 cell line.^[^ ^10^ ^]^ The PITPNM3‐mutated truncate cannot phosphorylate PTK2B as well as downstream factors protein tyrosine kinase (PTK) and SRC upon CCL18 treatment (Figure S1E, Supporting Information). It is confirmed PTK2B binding domain of PITPNM3 is responsible for PTK2B phosphorylation and signaling transduction. This makes it possible to target PITPNM3 by disrupting the PTK2B binding domain of PITPNM3 conducted PTK2B phosphorylation. Thus we designed a series of small molecules targeting the PTK2B binding domain of PITPNM3.

### 2.2 Screening of Small Molecular Compounds Targeting PITPNM3

**Figure 2:**
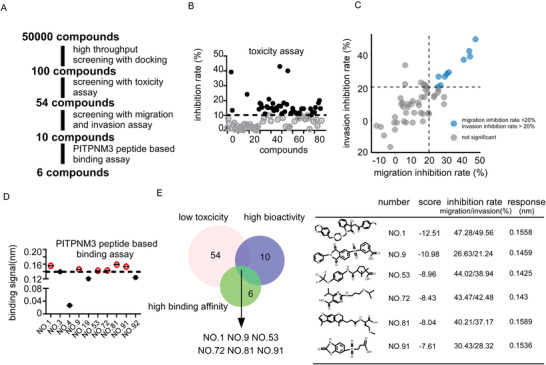
Screening of anti‐PITPNM3 compounds. A) Screening strategy of anti‐PITPNM3 compounds. B) Toxicity of top 100 small molecular compounds in silico scores. Compounds with inhibition rates below 10% were selected. C) Dot plot of migration as well as invasion inhibition rates of selected compounds. D) Binding signal of selected compounds confirmed by BioLayer Interferometry (BLI) assay. E) The intersection of compounds after three rounds of screening. Structures of leading compounds targeting PITPNM3 with their basic information.

Work flow of the screening PITPNM3 inhibitors is shown in **Figure** **2**A. To screen small molecular compounds targeting PITPNM3, we constructed structure model for PITPNM3 based on known homology templates because of the unavailability of its sufficient crystal structure. We searched for suitable templates by the similarity of amino acid sequences for the PTK2B‐binding domain in Protein Data Bank (PDB) databases. A recently published crystal structure (PDB ID:1Z88) was identified as the best template and was used to construct a static PITPNM3 model which was further evaluated for model health and identified with a druggable binding pocket. Then more than 50K molecules with high diversity in the structure were pre‐selected and were then docked into the defined druggable binding pocket of PITPNM3. Top 100 compounds with high in silico scores were designated as primarily virtual hits (Table S1, Supporting Information). The top 100 compounds with the highest docking scores were selected and tested for cell viability inhibition effects. Among them, 54 compounds barely inhibited cell viability. Since PITPNM3 inhibition had little impact on cancer cell proliferation or apoptosis, 54 compounds with less toxicity were selected for the next screening round (Figure 2B; Table S2, Supporting Information). We then performed migration and invasion inhibition screening assays. 17 compounds can inhibit CCL18‐PITPNM3 signaling mediated migration with their inhibition rate over 20% while 16 compounds can inhibit CCL18‐PITPNM3 mediated invasion with an inhibition rate of over 20% (Figure 2C; Figures S6, S7, Tables S1, S2, Supporting Information). In all, ten compounds showed higher bioactivity in inhibiting CCL18‐PITPNM3 signaling mediated cell migration and invasion (Figure S8A, Supporting Information). We assessed the PITPNM3 binding ability of these ten compounds through the BioLayer Interferometry (BLI) assay. The BLI assay can directly monitor the binding affinity as well as binding kinetics between small molecular compounds and target proteins in vitro. 4 of 10 compounds were discarded because their BLI response was less than 0.14 nm (Figure 2D; Figure S8B, Supporting Information). Besides, the difference in efficacy of these ten compounds was further confirmed by transwell migration and invasion assay (Figure S8C, Supporting Information). C8018‐7840 (NO.1) among these ten compounds showed the highest efficacy. Overall, 6 of 100 compounds were identified to exhibit low toxicity, high bioactivity, and high binding response, and the best compound C8018‐7840 was chosen as a leading compound (Figure 2E).

### 2.3 C8018‐7840 Treatment Modulates the PITPNM3 Signaling

**Figure 3:**
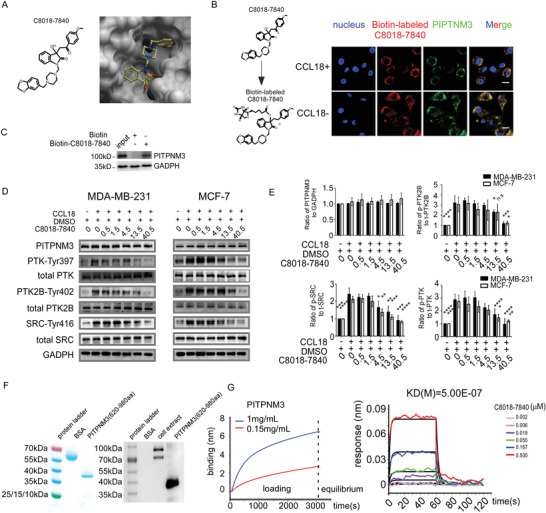
Anti‐PITPNM3 compounds inhibit metastasis signaling by targeting PITPNM3. A) The docking performance between C8018‐7840 (NO.1 compound) and the binding pocket of PITPNM3model. B) Co‐localization of Biotin‐C8018‐7840 and PITPNM3. Scale bar 10 µm. C) Biotin‐C8018‐7840 immunoprecipitation assay. PITPNM3 could be precipitated by Biotin‐C8018‐7840. D) C8018‐7840 inhibits CCL18‐PITPNM3 signaling pathways in a concentration‐dependent manner in MDA‐MB‐231 and MCF‐7. Cells treated with CCL18 at 100 ng mL^−1^ for 72 h. E) Three independent results of Western Blot of PITPNM3 signaling pathways. F) SDS page and Western Blot of purified PITPNM3 protein. G) Binding affinity between C8018‐7840 and purified human recombinant PITPNM3 analyzed by Biolayer interferometry (BLI) on the Fortebio Octet platform. All data were expressed as means with ±SD of three independent experiments and data was compared with the CCL18 treated group.*p < 0.05, **p < 0.01, ***p < 0.001,****p < 0.0001.

Docking performance between C8018‐7840 and PITPNM3 was shown in **Figure** **3**A. To confirm C8018‐7840 binding with PITPNM3, we synthesized a biotinylated derivative of C8018‐7840. Biotin‐C8018‐7840 can co‐localize with PITPNM3 in a CCL18‐independent manner (Figure 3B). Besides, to further confirm PITPNM3 binds with C8018‐7840, an immunoprecipitation (IP) was performed with biotin‐C8018‐7840 by using an antibody against Biotin. As shown in Figure 3C, endogenous PITPNM3 in MDA‐MB‐231 cells could be precipitated by biotin‐C8018‐7840. We next examined whether C8018‐7840 could block PITPNM3‐dependent signaling. Previous studies suggested that PITPNM3 phosphorylated PTK2B at Tyr402, PTK at Tyr397,and SRC at Tyr416 upon CCL18 treatment.^[^ ^3^ ^]^ Our results showed that phosphorylation of PTK2B Tyr402, PTK Tyr397, and SRC Tyr416 could be induced after CCL18 treatment (Figure 3D). Next, we found C8018‐7840 treatment inhibited phosphorylation of PTK2B at Tyr402, PTK at Tyr397, and SRC at Tyr416 in a concentration‐dependent manner and significantly abrogated their phosphorylation at 40.5 µm in MDA‐MB‐231 and MCF‐7 cells (Figure 3E). To determine the binding affinity of C8018‐7840, we purified recombinant PITPNM3 with a purity of over 95%. Through the SDS page and an antibody recognizing PITPNM3 C‐terminal peptide, the molecular weight of recombinant PITPNM3 was confirmed at ≈40 Kd (Figure 3F). The loading signal of target proteins is recommended to be over 4 nm to detect protein and small‐molecule binding by the manufacturer. The loading signal of PITPNM3 can reach 6.48 nm while the loading signal of CCL18 can reach 8.26 nm (Figure 3G). The binding affinity between C8018‐7840 and PITPNM3 show its *K*_D_ value of ≈500 nm (Figure 3G), while the binding affinity between C8018‐7840 and CCL18 is 0.11 m (Figure S9A, Supporting Information). To further confirm that C8018‐7840 is PITPNM3 dependent, we employed stable PITPNM3 knockdown MDA‐MB‐231 cells. C8018‐7840 significantly abrogated CCL18 treated vector cells while had little impact on the migration ability of stable PITPNM3 knockdown MDA‐MB‐231 cells (Figure S9B, Supporting Information). To confirm the selectivity and off‐target effects, we test the effect of C8018‐7840 on its potential targets. Epidermal growth factor (EGF) was known as PITPNM1 signaling activators.^[^ ^8^, ^11^ ^]^ EGF promoted MDA‐MB‐231 migration while C8018‐7840 cannot inhibit EGF‐PITPNM1 mediated migration (Figure S9C, Supporting Information). In addition, we acquired short hairpin RNA to knockdown PITPNM1, PITPNM2, as well as PTK2B, respectively. Knockdown of PITPNM1, PITPNM2, or PTK2B would suppress cell migration (Figure S9D–F, Supporting Information). PITPNM1, as well as PITPNM2 located in the cytoplasm, which was confirmed as previously reported (Figure S9G, Supporting Information),^[^ ^8^, ^12^ ^]^ while PITPNM3 and biotin‐C8018‐7840 are located on the membrane (Figure 3B). In addition, two greater EMT and metastasis inducer: TGF‐*β* and CXCL12, which are highly abundant in TME, were used to confirm the selectivity of C8018‐7840. TGF‐*β* and CXCL12 significantly promote MDA‐MB‐231 cell migration while C8018‐7840 had little impact on TGF‐*β* and CXCL12 induced cell migration (Figure S9C, Supporting Information). Other metastatic regulators such as: Cathepsin C (CTSC) and sphingosine‐1‐phosphate transporter spinster homologue 2 (SPNS2) were also validated.^[^ ^13^ ^]^ CTSC is highly expressed by MDA‐MB‐231 and SPNS2 is highly expressed by MDA‐MB‐231 and BT‐549 (Figure S9G, Supporting Information). Culture of breast cancers with tumor associated neutrophils (TANs) cultured medium promotes MDA‐MB‐231 migration and partly reversed by DNase I, while C8018‐7840 did not alter TANs induced cell migration (Figure S9H, Supporting Information). Besides, C8018‐7840 have no effect on CTSC induced TANs recruitment (Figure S9H, Supporting Information). As for SPNS2, knockdown of SPNS2 significantly promotes MDA‐MB‐231 cell migration which is accordant with previous study^[^ ^14^ ^]^ (Figure S9I, Supporting Information). SPNS2 seems to function as a breast cancer suppressor, not only it can inhibit breast cancer cell migration, but high level of SPNS2 predicts good survival in different cohorts (Figure S9I, Supporting Information). Taken together, C8018‐7840 is a potent PITPNM3 inhibitor without exceptional selectivity over CCL18, TGF‐*β*, CXCL12, PITPNM1, PITPNM2, or PTK2B.

### 2.4 C8018‐7840 Inhibits PITPNM3 Dependent Tumor Metastasis

**Figure 4:**
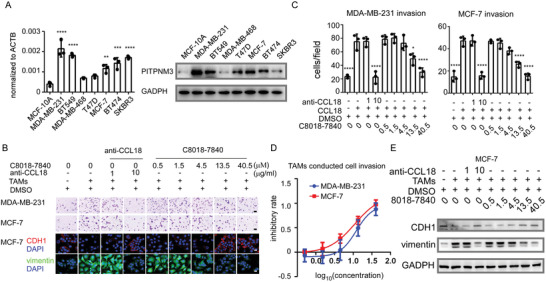
The effects of C8018‐7840 on PITPNM3 conducted metastasis in breast cancer cells. A) Expression of PITPNM3 in different breast cancer cell lines which are detected by qPCR and Western Blot. Data were compared with MCF‐10A. B) Representing images of anti‐invasion effects of C8018‐7840 on PITPNM3 conducted migration and invasion in MDA‐MB‐231 and MCF‐7 cells when co‐cultured with tumor associated with macrophages. Scale bar 100 µm. Representing images of anti‐EMT effects of C8018‐7840. Scale bar 20 µm. C) Statistical diagrams of anti‐invasion effects of C8018‐7840 on PITPNM3 conducted migration and invasion in MDA‐MB‐231 and MCF‐7 cells. D) EC50 anti‐invasion regression fitting curve of C8018‐7840 of MDA‐MB‐231 and MCF‐7 cells. E) Inhibition effects of C8018‐7840 on EMT in MCF‐7 cells which are validated by Western Blot. All data were expressed as means with ±SD of three independent experiments and data was compared with the CCL18 treated group.*p < 0.05, **p < 0.01, ***p < 0.001, ****p < 0.0001.

We next evaluated PITPNM3 inhibitor C8018‐7840 for their metastasis inhibitory activities in human breast cancer cell lines. PITPNM3 is much higher in MDA‐MB‐231, BT549, MCF‐7, BT474, and SKBR3 (**Figure** **4**A). As reported by our previous studies, TAMs secreted CCL18 and induced breast cancer cell invasion and EMT through PITPNM3.^[^ ^3^, ^9^, ^15^ ^]^ To explore the inhibitory effects of C8018‐7840 on TAMs‐conducted invasion, we first acquired TAMs. We treated monocyte‐derived macrophages and THP‐1‐derived macrophages with the conditioned culture medium of MDA‐MB‐231 cells according to our previous studies.^[^ ^15^ ^]^ Consistently, these TAMs exhibited elongated morphology, a CD206^high^ phenotype, and secreted CCL18 (Figure S10A,B, Supporting Information), which is in accord with our previous studies.^[^ ^15^ ^]^ Then, Boyden chambers were used to examine the anti‐invasion ability of C8018‐7840 in TAMs co‐culture systems. C8018‐7840 showed an significant inhibitory effect on TAMs‐conducted invasion in an concentration dependent manner and significantly abrogated cell invasion at 40.5 µm, while 10 µg mL^−1^ CCL18 neutralized antibody can inhibit TAMs conducted invasion as well (Figure 4B; Figure S10C, Supporting Information). In addition, we validated the effects of C8018‐7840 in hepatocellular carcinoma (SNU‐449 and PLC), pancreatic ductal carcinoma (PANC‐1 and SUIT‐2), and breast cancer (MDA‐MB‐231 and MCF‐7) under CCL18 treatment. C8018‐7840 inhibited PITPNM3‐induced cell migration as well as invasion in a concentration‐dependent manner in PANC‐1, SUIT‐2, PLC, and SNU‐449 (Figure S10D,E, Supporting Information). Interestingly, we found PANC‐1 showed less sensitivity to C8018‐7840. This could partly attribute to the high expression of PITPNM3 in PANC‐1 (Figure S5B, Supporting Information). Besides, we found that C8018‐7840 inhibited PITPNM3‐induced cell migration as well as invasion in a concentration‐dependent manner in MDA‐MB‐231 and MCF‐7 (Figure S11A–C, Supporting Information). In addition, we also tested the effects of C8018‐7840 on PITPNM3 conducted EMT in MCF‐7. Immunofluorescent, Western Blot, and qPCR showed that C8018‐7840 notably decreased CCL18‐PITPNM3 induced vimentin and recovered CDH1 expression (Figure 4B,E; Figure S11D,E, Supporting Information), which further confirmed that C8018‐7840 inhibited PITPNM3 dependent tumor metastasis.

### 2.5 C8018‐7840 Loaded Nanoparticles Showed Favorable Pharmacokinetics

**Figure 5:**
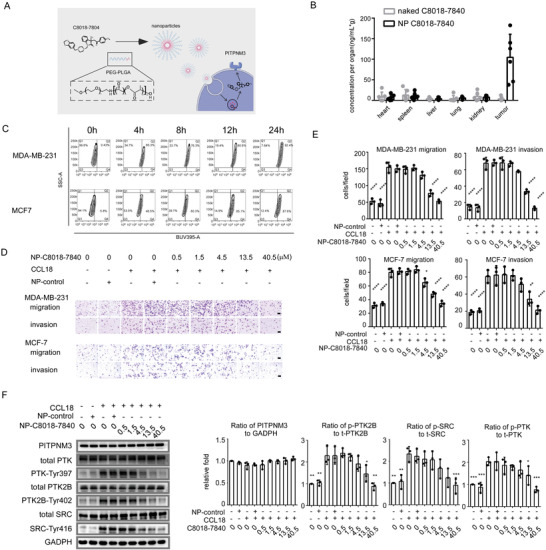
The in vitro effects of C8018‐7840 loaded nanoparticles (NPs). A) Schematic illustration of NPs for C8018‐7840 delivery and PITPNM3 inhibition. B) The accumulation of NP‐C8018‐7840 in tumor tissue, heart, liver, kidney, and spleen, which was detected by LC‐MS/MS after 24 h of NP‐C8018‐7840 of mice tail vein injection. C) Intake efficiency of MDA‐MB‐231 and MCF‐7 treated with NP‐C8018‐7840. D) Representing images of anti‐migration effects and anti‐invasion effects of NP‐C8018‐7840 in MDA‐MB‐231 and MCF‐7. Scale bar 100 µm. E) Statistical diagrams of anti‐migration effects and anti‐invasion effects of NP‐C8018‐7840 in MDA‐MB‐231. F) NP‐C8018‐7840 inhibits PITPNM3 signaling pathways. All data were expressed as means with ±SD of three independent experiments and data was compared with the CCL18 treated group. *p < 0.05, **p < 0.01, ***p < 0.001, ****p < 0.0001.

Most small molecular inhibitors are metabolized by the liver. Candidates targeting PITPNM3 were incubated with rat liver microsomes to determine whether they are metabolized by the liver. C8018‐7840 diminished before 20 mins, while NO.53, NO72, and NO.81 still remained at 77.01%, 92.96%, and 80.84%, respectively (Figure S12A, Supporting Information). To assess the utility of C8018‐7840 in animal experiments, the pharmacokinetics of C8018‐7840 was tested. C8018‐7840 exhibited poor pharmacokinetic profiles following p.o. or i.v. administration (Figure S12B, Supporting Information). C8018‐7840 barely absorbed by oral administration. The poor oral bioavailability could be attributed to the solubility of compounds in the gastrointestinal fluid, low stability in the gastrointestinal environment and poor gastrointestinal cells membrane permeability.^[^ ^16^ ^]^ During preparation, we found that C8018‐7840 is stable in acid environment but unstable in alkaline environment, which the poor oral bioavailability might be attributed to the solubility of compounds in the gastrointestinal fluid and poor gastrointestinal cells membrane permeability, as solubility of C8018‐7840 is around 50 mg L^−1^, which is a hard‐to‐dissolve compound. The half‐lives were observed for 30.6 minutes when dosed at 5 mg kg^−1^ i.v. administration. In recent years, nanoparticles (NPs) based drug delivery systems have been proved to be promising for cancer drug delivery because they can increase half‐lives of vulnerable drugs and allow targeted release in tumor sites.^[^ ^17^ ^]^ Poly ethylene glycol (PEG) and poly lactic‐*co*‐glycolic acid (PLGA) are US Food and Drug Administration (FDA) approved synthetic polymers. To improve the pharmacokinetics of C8018‐7840, we use polyethylene glycol‐modified poly lactic‐*co*‐glycolic acid (PEG‐PLGA) nanoparticle for C8018‐7840 delivery (**Figure** **5**A). Through excitation‐emission and absorbance scan, C8018‐7840 exhibited a specific fluorescence in a concentration‐dependent manner at 390–410 nm excitation wavelength but showed no response to absorbance scan (Figure S12C, Supporting Information). Based on this, we constructed a weight and fluorescence intensity regression equation to calculate the loading rate (Figure S12D, Supporting Information). We used different concentrations of C8018‐7840 to prepare C8018‐7840 loaded nanoparticle (NP‐C8018‐7840) with its volume ratio to PEG‐PLGA at 1:20. Their load rates were shown in Figure S12E, Supporting Information. NP‐C8018‐7840 could be formed with an average size of 83.38 nm and a zeta potential of −17.9 mV (Figure S12F, Supporting Information).

After successful preparation of NP‐C8018‐7840, we next investigate whether these NPs would show good pharmacokinetics. Of great importance, NP‐C8018‐7840 exhibited prolonged pharmacokinetics with half‐lives observed 2.05 h following i.v. administration (Figure S12B, Supporting Information). In addition, Balb/c nude mice were used to investigate the biodistribution of NP‐C8018‐7840. After 24 h of intravenous injection, LC‐MS/MS confirmed that C8018‐7840 loaded nanoparticles showed its accumulation in the tumor tissue than in other tissues including heart, liver, kidney, spleen, as well as lung and than that of naked C8018‐7840 in tumor with a 5 mg kg^−1^ dosage and C8018‐7840 was still detectable in tumor after 48 h injection (Figure 5B; Figure S12G, Supporting Information). Besides, after six times of injections, no noticeable histological toxicity of NP‐C8018‐7840 was observed in the heart, liver, spleen, lung, and kidney (Figure S13A, Supporting Information). Next, we investigate whether NP‐C8018‐7840 could efficiently inhibit PITPNM3 conducted metastasis. By using a 395 nm channel containing flow cytometry, it is confirmed that NP‐C8018‐7840 can be taken in by MDA‐MB‐231 and MCF‐7 cells at 24 h (Figure 5C). NP‐C8018‐7840 abrogated PITPNM3 conducted migration as invasion in MDA‐MB‐231 and MCF‐7 in a concentration‐dependent manner (Figure 5D,E). NP‐C8018‐7840 significantly inhibited phosphorylation of PTK2B at Tyr402, PTK at Tyr397, and SRC at Tyr416 in a concentration‐dependent manner like naked C8018‐7840 (Figure 5F). These results demonstrate that C8018‐7840 loaded nanoparticles exhibited favorable pharmacokinetics and can inhibit PITPNM3 dependent tumor metastasis as well.

### 2.6 NP‐C8018‐7840 Inhibits PITPNM3 Driven Tumor Metastasis In Vivo

**Figure 6:**
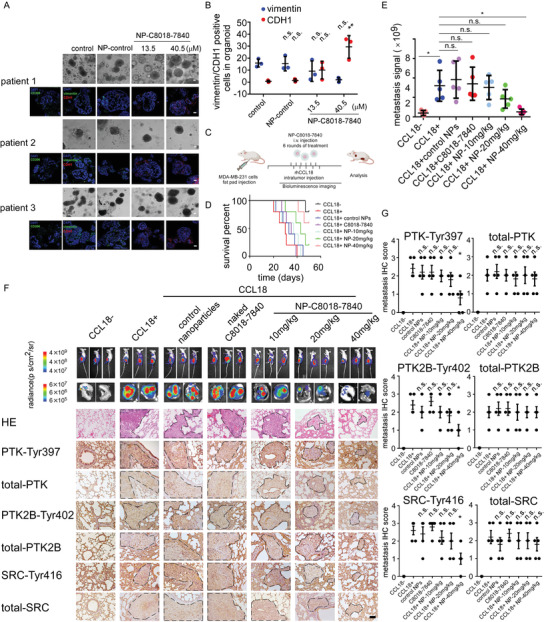
NP‐C8018‐7840 inhibits distant metastasis in vivo. A) Images of patient‐derived organoids treated with NP‐C8018‐7840 and vimentin as well CDH1 expression which is detected by immunofluorescence. Scale bar for light microscope 100 µm. Scale bar for immunofluorescence 30 µm. B) Statistical diagrams of vimentin as well CDH1 expression in patient‐derived organoids treated with NP‐C8018‐7840. C) The dosing cycle of NP‐C8018‐7840 injection in animal study. D) Kaplan–Meier survival curve for the mice‐bearing MDA‐MB‐231 xenografts. E) Statistical diagram of chest bioluminescence of mice bearing MDA‐MB‐231‐Luc orthotopic tumor. F) Images of mice bearing MDA‐MB‐231‐Luc orthotopic tumor. Bioluminescence images of lungs harvested from mice bearing MDA‐MB‐231‐Luc orthotopic tumor. H&E staining and IHC staining of phosphorylation PTK2B at Tyr402, PTK at Tyr397,SRC at Tyr416, total‐PTK2B, total‐PTK, and total‐SRC of lungs harvested from mice bearing MDA‐MB‐231‐Luc orthotopic tumor. All data were expressed as means with ±SD of three independent experiments and data was compared with the CCL18 treated group. G) Statistical diagrams of IHC staining of phosphorylation PTK2B at Tyr402, PTK at Tyr397,SRC at Tyr416, total‐PTK2B, total‐PTK, and total‐SRC of lungs harvested from mice bearing MDA‐MB‐231‐Luc orthotopic tumor. Scale bar 40 µm. *p < 0.05, **p < 0.01, ***p < 0.001, ****p < 0.0001.

Patient‐derived organoids (PDOs), which show a similarity to original tumors, can retain TME stromal components.^[^ ^18^ ^]^ PDOs are a robust preclinical model for investigating therapy sensitivity and a sufficient tool to study metastasis.^[^ ^19^ ^]^ We employ breast cancer PDOs to explore the anti‐metastasis effects of NP‐C8018‐7840. After ten days of incubation, CD206 positive cells can be detected in the breast cancer organoid (**Figure** **6**A). 40.5 µm of NP‐C8018‐7840 significantly abrogates the expression of vimentin while promoting CDH1 expression in PDOs (Figure 6A,B). Then we evaluated the anti‐metastasis potential of NP‐C8018‐7840 in vivo by using mouse xenograft tumor models. Luciferase expressed MDA‐MB‐231 cells (MDA‐MB‐231‐Luc) and wild‐type MDA‐MB‐231 cells were injected orthotopically in the fat pad of Balb/c nude mice. Recombinant CCL18 was injected intratumor at a dosage of 0.1 µg kg^−1^ biweekly. Anti‐metastasis effects of NP‐C8018‐7840 were further examined in mice bearing MDA‐MB‐231‐Luc as well as MDA‐MB‐231 orthotopic tumors. Considering pharmacokinetics and biodistribution of NP‐C8018‐7840, the initial test dosage was set to 10 mg kg^−1^ and was injected through the mice's tail vein every 48 hrs 6 times (Figure 6C). Mice bearing MDA‐MB‐231 orthotopic tumors treated with NP‐C8018‐7840 exhibited a prolonged overall survival (Figure 6D). In addition, nude mice with MDA‐MB‐231‐Luc orthotopic tumors were monitored by bioluminescence. Of note, when compared with the CCL18 treatment and CCL18 plus control nanoparticles treatment, the bioluminescence metastasis signal of mice significantly dropped in the 40 mg kg^−1^ NP‐C8018‐7840 group in a dose‐dependent manner (Figure 6E). When the experiment was terminated, lungs in each group were dissected and examined for metastasis. Bioluminescence signal for harvested lungs showed that metastasis was inhibited by NP‐C8018‐7840 treatment (Figure 6F). Hematoxylin and eosin staining (H&E) also showed that the group treated with CCL18 or CCL18 plus control nanoparticles exhibited lung metastasis and groups treated with NP‐C8018‐7840 displayed limited lung metastatic propensity (Figure 6F). Besides, to further evaluate whether NP‐C8018‐7840 inhibits in vivo metastasis by blocking PTK2B signaling pathway, we performed IHC for phosphorylation PTK2B, PTK, and SRC. IHC staining showed that 20 and 40 mg mL^−1^ NP‐C8018‐7840 can inhibit the phosphorylation of PTK2B at Tyr402, PTK at Tyr397, and SRC at Tyr416, but did not alter the level of total‐PTK2B, total‐PTK, and total‐SRC in vivo (Figure 6F,G). The primary tumor was also tested through IHC, NP‐C8018‐7840 significantly inhibited the phosphorylation of PTK2B at Tyr402, PTK at Tyr397, and SRC at Tyr416 of the breast cancer primary site, but had little impact on total‐PTK2B, total‐PTK, and total‐SRC (Figure S13B, Supporting Information).

## 3 Discussion

Current therapeutic strategies aim to abrogate tumor growth and eradicate cancer cells. Most therapeutics rely on their cytotoxic, DNA‐damage, and cell cycle arrest capability to eliminate cancer. However, cancer cells often acquired the ability to invade cell death programs caused by therapeutic agents and consistently developed relapse or distant metastasis. Given the fact that cancer metastasis accounts for the majority of breast cancer deaths, we focus on understanding the molecular mechanisms underlying breast cancer metastasis and developing small molecules to reverse breast cancer metastasis.

PITPNM3 has been demonstrated as a druggable target because it had been discovered to promote breast cancer,^[^ ^3^ ^]^ hepatocellular carcinoma^[^ ^5a^ ^]^ and pancreatic ductal carcinoma^[^ ^6a^ ^]^ metastasis and our systematic analysis revealed its oncogenic roles in pan‐cancer. PITPNM3 interacts with PTK2B through its PTK2B binding domain. In the tumor microenvironment, TAMs‐derived CCL18 binds to PITPNM3. After binding, PITPNM3 will phosphorylate PTK2B via PTK2B binding domain which leads to metastasis‐related signaling pathways activation.^[^ ^3^, ^7^, ^9^ ^]^ This process promotes cancer metastasis, on the other hand, transforms cancer cells into mesenchymal phenotype. The mesenchymal‐phenotype cancer cells will secret several kinds of cytokines and chemokines to transform macrophages into an M2‐like phenotype which forms a positive feedback loop.^[^ ^15^ ^]^ PITPNM3 inhibition could break the bad loop and reverse cancer metastasis. Therefore, oncogenic PITPNM3 is considered a potential target for reversing cancer metastasis.

To date, some therapeutic agents targeting CCL18 and PTK2B have been reported. A small molecular inhibitor, NVP‐TAE 226, was found to be an inhibitor of PTK and IGF1R. It can also inhibit PTK2B as well, but this small molecular compound seems to be a non‐selective tyrosine kinase inhibitor that can inhibit fibroblast growth factor receptor (FGFR), platelet derived growth factor receptor (PDGFR), insulin receptor (INSR), and Cyclin dependent kinase 1 (CDK1) as well.^[^ ^20^ ^]^ Based on computer‐aided virtual screening and anti‐migration assay in vitro, another study reported 15 small molecules which interacted with CCL18 and blocked CCL18‐PITPNM3 interactions.^[^ ^21^ ^]^ In addition, CCL18 neutralized antibody for basic experimental research had also been reported.^[^ ^3^, ^22^ ^]^ However, it is still urgent to develop inhibitors to block PITPNM3, because CCL18 had been shown to trigger biological responses in T cells, B cells, dendritic cells, hematopoietic progenitor cells, and fibroblast under normal conditions,^[^ ^23^ ^]^ and this raises the possibilities that therapeutics agents targeting CCL18 might result in adverse effects such as deficiency of T cells or B cells. Therefore, we aim to discover small molecular inhibitors targeting PITPNM3. Through molecular docking and three rounds of high throughput screening, six compounds potentially antagonizing PITPNM3 out of 50 000 compounds library were selected. These compounds exhibited higher anti‐migration as well as anti‐invasion bioactivity, higher docking score and higher binding response but lower toxicity. Among these six compounds, C8018‐7840 showed the most potent affinity with K_D_ at in the micromolar range and could significantly inhibit PITPNM3 conducted migration, invasion, and signaling pathway activation in vitro and in vivo.

Our study has several important implications. It provided the very first example of small molecules targeting PITPNM3. Through homology modeling and structure‐based virtual docking, we overcome the difficulty of the unavailability of the crystal structure of PITPNM3 and turn PITPNM3 into a druggable target. Besides, these PITPNM3 inhibitors exhibit PITPNM3‐specific inhibition and reduce metastasis of breast cancer cells. This provided new small molecular inhibitors to reverse PITPNM3‐related metastasis of different cancers and potentially break the bad feedback loop between tumor microenvironment and tumor. Although C8018‐7840 showed a poor pharmacokinetic with its short half‐lives, we constructed a C8018‐7840 loaded nanoparticles and NP‐C8018‐7840 significantly abrogates cancer distant metastasis in vivo. It provides a promising future for nanoparticle‐based drug delivery.

## 4 Experimental Section

The materials and methods used in this study, including cell lines, chemicals, antibodies, and molecular docking, peptide synthesis, binding assay, MTT assay, transwell assay, Western Blot, qPCR, nanoparticle preparation, and organoid preparation are described in Supporting Information.

## Conflict of Interest

The authors declare no conflict of interest.

## Supporting information

Supporting Information

## Data Availability

The data that support the findings of this study are available in the supplementary material of this article.
