# Supplementary material for: Small Molecular Inhibitors Reverse Cancer Metastasis by Blockading Oncogenic PITPNM3
Source: Adv Sci (Weinh). 2022 Oct 26;9(35):2204649. doi: 10.1002/advs.202204649 (PMC9762305; doi:10.1002/advs.202204649)
Supplement: Supplementary file 1 — Supporting Information [file ADVS-9-2204649-s001.pdf]

ADVANCED SCIENCE
Open Access

Supporting Information

for *Adv. Sci.*, DOI 10.1002/advs.202204649

Small Molecular Inhibitors Reverse Cancer Metastasis by Blockading Oncogenic PITPNM3

Zihao Liu, Yu Shi, Li Lv, Jianing Chen, WenG. Jiang, Jun Li, Qun Lin, Xiaolin Fang, Jingbo Gao, Yujie Liu, Qiang Liu, Xiaoding Xu, Erwei Song* and Chang Gong**

Supplementary information for

Small molecular inhibitors reverse cancer metastasis by blockading oncogenic PITPNM3

Zihao Liu[1,2,6,]*, Yu Shi[1,2,]*, Li Lv[2,5,]*, Jianing Chen[1,2,]*, WenG. Jiang[3], Jun Li[4], Qun Lin[1,2], Xiaolin Fang[1,2], Jingbo Gao[1,2], Yujie Liu[1,2], Qiang Liu[1,2], Xiaoding Xu[2,#], Erwei Song[1,2,#] , Chang Gong[1,2,#]

[1] Breast Tumor Center, Sun Yat-sen Memorial Hospital, Sun Yat-sen University, Guangzhou, China

[2] Guangdong Provincial Key Laboratory of Malignant Tumor Epigenetics and Gene Regulation, Sun Yat-sen Memorial Hospital, Sun Yat-sen University, Guangzhou 510120, China

[3] Cardiff China Medical Research Collaborative, School of Medicine, Cardiff University, Heath Park, Cardiff CF14 4XN, UK

[4] Department of Biochemistry, Zhongshan School of Medicine, Sun Yat-sen University, Guangzhou 510080, China

[5] Department of Pharmacy, Sun Yat-Sen Memorial Hospital, Sun Yat-Sen University, Guangzhou, 510120, China

[6] Department of Breast and Thyroid Surgery, The Second Clinical Medical College of Jinan University, The First Affiliated Hospital of Southern University of Science and Technology, Shenzhen People's Hospital, Shenzhen, 518020, China.

*These authors contribute equally to this work. [#] Correspondence

Correspondence authors: Chang Gong (E-mail: gchang@mail.sysu.edu.cn or changgong282@163.com); Erwei Song (E-mail: songew@mail.sysu.edu.cn), Xiaoding Xu (E-mail: xuxiaod5@mail.sysu.edu.cn)

This PDF file includes:

Supplementary text

Figure S1-S13

Table S1-S4

Supplementary Experimental Procedures

Cell culture

Human breast cancer cell line MDA-MB-231 and MCF-7, human immortalized non-tumorigenic mammary epithelial cell line MCF-10A, human immortalized embryonic kidney cell line HEK-293Tand human myeloid leukemia mononuclear cell line THP-1 were purchased from ATCC. All cell lines were certificated by short tandem repeat (STR) analysis, passaged less than 6 months, and cultured according to the recommended protocols. Briefly, MDA-MB-231, MCF-7, and HEK-293T were grown in Dulbecco's modified Eagle medium which contains 10% fetal bovine serum. MCF-10A were grown in Dulbecco's modified Eagle medium/F12 which containing 5% horse serum, epidermal growth factor 50ng/ml, insulin 10μg/ml and hydrocortisone 0.5μg/ml

Molecular docking and compound library

The homology modeled structure was used as templates to perform molecular docking virtual screening against small molecule database. More than 50K molecules with high diversity in structure were pre-selected from the ChemDiv compound database. The selected structures were then docked into the defined binding site using a static protein mode. The docking procedure was performed by employing MOE software. The 354 amino acid sequence of the human PITPNM3 C-terminal PTK2B-binding domain from 620 to 974 was obtained from the National Center for Biological Information (NCBI). The sequence was BLASTed against templates amino acid sequences from Protein Data Bank (PDB) database. The best template was identified and selected by E-value sequence for identity and coverage. The target amino acid sequence was aligned to the best templates by default settings through the Clustal Omega web server tool (European Bioinformatics Institute, EMBL-EBI). The homology model was constructed in ICM-Pro 3.8 software (MolSoft). Besides, the short motifs within the PITPNM3 C-terminal PTK2B-binding domain were predicted by the EMBL-EBI InterPro web server.

Peptide synthesis

Recombinant his tag fusion PITPNM3 protein was prepared by expression of PITPNM3 peptides cloned into the pET-22b vector with an N-terminal His tag. pET-22b-PITPNM3 plasmids were transformed into E.coli (BL21). Bacterial cells were cultured at 1000r.p.m 37 ° C to an OD600 of 0.6 and were treated with IPTG (isopropyl 1-thio-beta-D-galactopyranoside) to induce the expression of recombinant fusion protein at 37 ° C for 3 hours. The bacterial cultures were certificated to collect bacterial cell pellets which are then re-suspended in lysis buffer (150mM NaCl, 10mM NaH_2PO_4, 5mM DTT, 1mM EDTA, pH 8.0) in the presence of protease inhibitor (protease inhibitor cocktail and 100μm PMSF). The suspension was then sonicated 4 ° C. The PITPNM3 fusion protein was purified by His Trap HP Column kit (GE Healthcare) as instructed. The eluted proteins were then centered using an ultrafiltration centrifugal tube (Millipore) and stored by eluting with PBS.

Kinetics and affinity binding assay

The biotinylated recombinant PITPNM3 was prepared using a commercial biotinylation kit (BioMedia, G-MM-IGT). Generally, stock protein without free amine was mixed with freshly

prepared biotin buffer with a 1:1 molar ratio and incubated at room temperature for 1 hour. The mixture was transferred into the desalting column to remove the unreacted free biotin reagent. The kinetics and binding affinity between small molecular compounds and PITPNM3 were determined using Biolayer interferometry (BLI) on the Fortebio Octet platform (USA). PBS with 1%DMSO and 0.01% Tween-20 was used as the assay buffer. The biotin-labeled PITPNM3 was tethered on Super Streptavidin (SSA) senor (Fortebio) with the saturation response level reaching 4 nm. Sensors with PITPNM3 tethered were equilibrated for 5 min to eliminate nonspecific bounding protein and establish baselines. Wash-association-dissociation cycles of compounds begin at the lowest compound concentration. DMSO only PBS buffer was used as reference. Raw kinetics data were collected and processed in the analysis software provided by the manufacturer. The data were analyzed through a 1:1 binding model and based on kon and koff, kinetics (KD) and affinity (steady-state KD) were calculated.

Transwell assay

Transwell assay was performed according our previous study[1]. Migration and invasion of breast cancer cells examined using Boyden chambers (Corning). For Matrigel coated assay, the upper chamber of the 8 μm inserts were coated with Matrigel (Corning) which is diluted with PBS. After incubation with stimuli, breast cancer cells (10^5 cells/well) were suspended with serum-free culture medium and plated in the upper chambers of the inserts (uncoated inserts for migration assay and coated inserts for invasion assay). After cultured for 12 to 24 hours for MDA-MB-231 and 36 to 48 hours for MCF-7 at 5% CO_2 37° C, cells which did not migrate or invade were removed. The inserts then were fixed with 4% formaldehyde, stained with crystal violet and counted cells per field under microscope.

MTT assay

MTT assay was performed by MTT assay kit. Briefly, MDA-MB-231 cells were seeded in 96-well plates and incubated with small molecular compounds at single concentration (5μM) for 48 hours. Then cells were washed with PBS for three times and incubated with MTT solution (5mg/mL) for 4 hours at 5% CO_2 37° C. After incubated with MTT solution, culture medium was discarded and 100μL DMSO was added to each well. The absorbance at 490nm was measured by microplate reader (TECAN spark)

Lentiviral preparation and transduction

The oligonucleotides encoding sh-PIPTNM3 or negative control were synthesized and cloned into the AgeI-EcoRI site of lentiviral vector pLKO.1. The oligonucleotide sequences were provided in supplementary table 2. For lentiviral preparation, HEK-293T (5×10^5) were transfected with 10ug sh-PIPTNM3- pLKO.1, 10ug psPAX2 and 5ug pMD2.G for 24 hours. After 24 hours culture, cells were incubated with new fresh culture medium for another 48 hours. The viral supernatant were collected and incubated with breast cancer cells which are then selected by puromycin. The transfection efficiency were further analyzed by qPCR.

Enzyme-linked immunosorbent assay (ELISA)

CCL18 levels in the culture medium of TAM-like macrophages were measured by Enzyme-linked immunosorbent assay (ELISA) assay using a commercial kit (BioVision,

E4519-100). Briefly, standard as well as samples is prepared as instructed. The ELISA plates were washed 2 times with wash buffer. The samples as well as standard were added into appropriate cells and incubate at 37° C for 1.5 hours. The content of plates were removed and then followed by adding antibodies and wash solution. HRP-streptavidin conjugate working solution and TMB substrate were added and used to detect CCL18 levels.

Western blotting

Proteins were extracted using RIPA lysis buffer in which PMSF, protease inhibitor cocktail and phosphatase inhibitor cocktails were added. Denatured protein extracts were loaded in wells of 8%-12% SDS-PAGE, transferred to 0.2μm pore size PVDF and labeled with antibodies against human Src (CST, 2109T, 1:100), PTK (CST, 71433T, 1:100), PTK2B (GeneTex, GTX130887, 1:100) and the corresponding phosphorylated forms p-Src-Tyr416(CST, 6943T, 1:100), p-PTK-Tyr397(CST, 8556T, 1:100), p-PTK2B-Tyr402 (CST, 3291S, 1:100), PITPNM3 (Novus, NBP1-31070, 1:100), GADPH (CST, 5174T, 1:200). HRP-linked anti-rabbit or anti-mouse IgG was used as secondary antibodies.

Quantitative real-time PCR

The relative expression of genes was analyzed using quantitative real-time PCR (qPCR). Briefly, total RNA was isolated using TRIzol (Life Technologies) according to the manufacturer's procedure. Complementary DNA (cDNA) was synthesized using the reverse-transcription kit (Vazyme, China) at 37° C for 15 minutes and 85° C for 5 minutes. The qPCR was performed in LightCycler 480 (Roche) using SYBR green mix reagent (Vazyme, China) according to the manufacturer's protocol. The reactions were done in a 10μl volume and performed in 40 cycles of PCR amplification at 95° C for 5s and 60° C for 30s following an 95° C initial stage. The relative expression of genes was calculated by the 2-ΔΔCt method which is normalized to ACTB. The primer sequences were provided as follow:

Gene	Forward primer	Reverse primer
ACTB	5'-TCATGAAGTGTGACGTGGACATC-3'	5'-CAGGAGGAGCAATGATCTTGATCT-3'
PITPNM3	5'-CAAGCAGCAATCAGGTAGGA-3'	5'-CAACTTAACCGGACAGACAAA-3'
PITPNM1	5'-GAGGAGTCTAGTGGTGAGGGC-3'	5'-TTCGGGTGTAGGGGTAGGC-3'
PITPNM2	5'-AAGACAGAAGAGGACCCCAAGC-3'	5'-CAGTAGCGGAACTCCACCTTG-3'
CDH1	5'-ATTTTTCCCTCGACACCCGAT-3'	5'-TCCCAGGCGTAGACCAAGA-3'
vimentin	5'-GCTCGTCACCTTCGTGAATA-3'	5'-CAGAGGGAGTGAATCCAGATTAG-3'
PTK2B	5'-AGATTCCCGACGAAACCC-3'	5'-GACACCTTCATAGACCTCCC-3'

Immunofluorescence

Cells were fixed with paraformaldehyde and washed with PBS for three times. Then cells were incubated with 5% BSA for 1 hour and incubated with primary antibodies against CD68 (GeneTex, GTX41865, 1:50), PITPNM3 (Novus, NBP2-33894, 1:50), PITPNM1 (ATLAS, HPA060227), PITPNM2 (ATLAS, HPA003414) and biotin (USA, Vector Laboratories) at 4° C for 24 hours, followed by washing with PBST for three times and incubating with Alexa594-linked or Alexa488-linked goat antibodies (CST) against mouse or rabbit IgG at room temperature for 1 hours. Then, cells were counterstained with DAPI and visualized

using confocal laser-scanning microscope (Carl Zeiss). As for organoid immunofluorescence, orgnoids were collected, embedded with OCT and subsequently analyzed through frozen section. Antibodies against CDH1(ABclonal, A3044) and vimentin (ABclonal, A19607) were used.

Flow cytometry

To evaluate the cell surface CD208 expression of induced TAMs-like cells, peripheral blood mononuclear cell and THP-1 cell line were treated with MDA-MB-231 culture medium. The induced cells were detached using EDTA trypsin and washed using PBS. Then cells were incubated with APC-labeled anti-human rabbit IgG antibody (Biolegend, 321109, 5μL per million cells in 100μL) at room temperature for 30 min, washed with PBS and analyzed in a C6 flow cytometry analyzer (BD Accuri). As for nanoparticles intake efficiency, cells were treated nanoparticles and harvested at different time points. Then cells were washed and analyzed in flow cytometry at excitation 395nm channel.

Hematoxylin and eosin staining (H&E) and Immunohistochemistry (IHC)

Paraffin embedded breast cancer tissue and paraffin embedded lungs collected from animal study were used for hematoxylin and eosin staining (H&E) and immunohistochemistry (IHC). For IHC, VECTASTAIN Elite ABC-HRP kit (USA, VectorLabs, PK-6200) was used according to the manufacturer’s instruction. Briefly, samples were deparaffinized, rehydrated, retrieved antigen, incubated with antibodies and HRP staining. Antibodies against human Src (Immunoway, YT1140, 1:100), p-Src-Tyr416 (CST, 2101S, 1:100), PTK (CST, D2R2E, 1:100), p-PTK-Tyr397 (Immunoway, YP0739,1:100), PTK2B (CST, 3292S, 1:100) and p-PTK2B-Tyr402 (CST, 3291S, 1:100) were used. For HE staining, samples were deparaffinized, rehydrated and stained with hematoxylin as well as eosin.

Nanoparticles preparation

Nanoparticles were prepared according to our laboratory protocols[2]. Briefly, a mixture of C8018-7840 (40mg/mL, in DMSO) and PEG5k-PLGA5k (20mg/mL, in DMF) in an volume ration of 1:20 was prepared. The mixture was added dropwise to 5mL sterilized deionized waster under stirring at 1000r.p.m. Then suspension was transferred into an ultrafiltration tube (Millipore, MWCO 100K) and centrifuged at 2800 r.p.m to removed free compounds as well as free organic solution. Then the nanoparticles (NPs) were collected and re-suspended in PBS. Size and zeta potential were examined by dynamic light scattering (Malvern, USA). To decide C8018-7840 encapsulation efficiency, freeze-dried NP-C8018-7840 was mixed with DMSO and detected the emission fluorescence intensity at 340nm excitation and 404 nm emission in microplate reader (TECAN spark). The encapsulation efficiency was calculated according the standard curve.

Organoid

Patient derived organoids were prepared according previous published studies[3]. Briefly, breast cancer tissues were washed with DMEM/F12 containing antibiotics, minced through sterilized scissors and digested with 2mg/mL collagenase on an shakers at 37° C for 3 hours. The suspension was strained over a 100μm sterilized filter while the retained tissue pieces

re-entered digested/shaking procedure. Then the filtered suspension was centrifugated at 800 r.p.m. The pellet was lysed with red blood cell lysis buffer for 10 minutes according to the manufacturer's protocol (China, Solarbio) and washed with PBS for three times. Then the pellet was incubated with ogranoid culture medium. The culture medium was prepared according to published study[3a].

Animal study

The animal experiments were performed at Sun Yat-Sen University Laboratory Animal Center (Guangzhou China). 4-weeks-old female BALB/c nude mice were purchased from Sun Yat-Sen University Laboratory Animal Center. All animal care and animal experimental use protocols were approved by the Institutional Animal Care and Use Committee of Sun Yat-Sen University (approval number: SYSU-IACUC-2020-000595) and were following national ethical guidelines for the care and maintenance of laboratory animals. For the orthotopic transplantation model, MDA-MB-231-Luc cells (10^6) were washed by PBS, re-suspended in PBS, and injected into the fourth mammary fat pads of the mice. Recombinant human CCL18 was injected intratumorally every three days (0.1μg/kg) for 2 weeks. Nanoparticle wrapped C8018-7840 were injected every 2 days for 6 times at 10mg/kg, 20mg/kg and 40mg/kg before CCL18 injection. The metastasis was examined using IVIS Lumina Imaging System. The lung was collected for further examination.

Bioinformatics analysis

The transcriptome profiling data was downloaded and merged by TCGAbiolinks[4]. TPM methods normalized transcriptome profiling data was further used for GSEA analysis. GSEA analysis was performed by GSEA software[5]. Hallmark gene sets and KEGG pathway gene sets were downloaded from (https://www.gsea-msigdb.org/gsea/).

Statistical analysis

All the statistical analyses were performed using GraphPad Prism 5 software. All experiments were repeated independently 3 times. For comparing normally distributed continuous variables between different groups, two-tailed Student's *t*-test or one-way ANOVA with Tukey's post hoc test were used. Kaplan-Meier plot with Log-rank test was used for survival analysis. $P < 0.05$ was considered statistically significant.

Reference for Supplementary methods:

[1] Z. Liu, Y. Zhou, G. Liang, Y. Ling, W. Tan, L. Tan, R. Andrews, W. Zhong, X. Zhang, E. Song, *Cell death & disease* **2019**, *10* (2), 1.

[2] a) C. Lin, Y. Tao, P. E. Saw, M. Cao, H. Huang, X. Xu, *Chemical Communications* **2019**, *55* (93), 13987; b) P. E. Saw, H. Yao, C. Lin, W. Tao, O. C. Farokhzad, X. Xu, *Nano letters* **2019**, *19* (9), 5967.

[3] a) N. Sachs, J. de Ligt, O. Kopper, E. Gogola, G. Bounova, F. Weeber, A. V. Balgobind, K. Wind, A. Gracanin, H. Begthel, *Cell* **2018**, *172* (1-2), 373; b) G. Vlachogiannis, S. Hedayat, A. Vatsiou, Y. Jamin, J. Fernández-Mateos, K. Khan, A. Lampis, K. Eason, I. Huntingford, R. Burke, *Science* **2018**, *359* (6378), 920.

[4] A. Colaprico, T. C. Silva, C. Olsen, L. Garofano, C. Cava, D. Garolini, T. S. Sabedot, T. M. Malta, S. M. Pagnotta, I. Castiglioni, *Nucleic acids research* **2016**, *44* (8), e71.

[5] A. Subramanian, P. Tamayo, V. K. Mootha, S. Mukherjee, B. L. Ebert, M. A. Gillette, A. Paulovich, S. L. Pomeroy, T. R. Golub, E. S. Lander, *Proceedings of the National Academy of Sciences* **2005**, *102* (43), 15545.

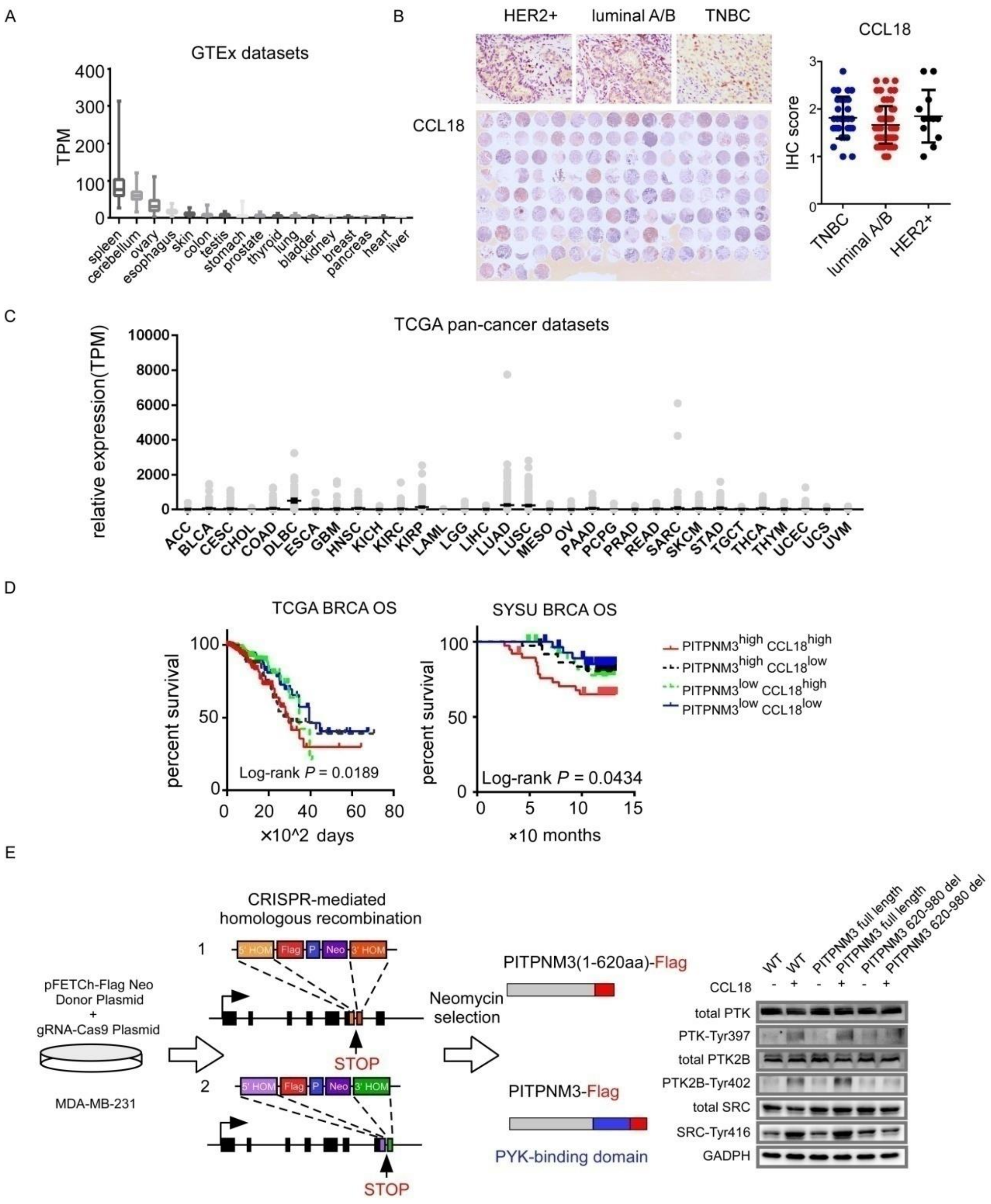

Supplementary Figure S1. Clinical implication of CCL18-PITPNM3 signaling pathway. A. The relative expression of PITPNM3 of different tissues in GTEx datasets. B. Represent images of CCL18 of breast cancer (BRCA) patients from SYSU memorial hospital, which expression was confirmed by IHC. C. The expression of CCL18 in different types of cancer from TCGA datasets. D. Co-expression of CCL18 and PITPNM3 predicts prognosis of breast cancer patients in TCGA BRCA and SYSU BRCA. E. Construction of PITPNM3(620-980) ^{del} cells by CRISPR-Cas9 method and the effect of the deletion of PYK2 binding domain of PITPNM3 on CCL18-PITPNM3 signaling pathway.

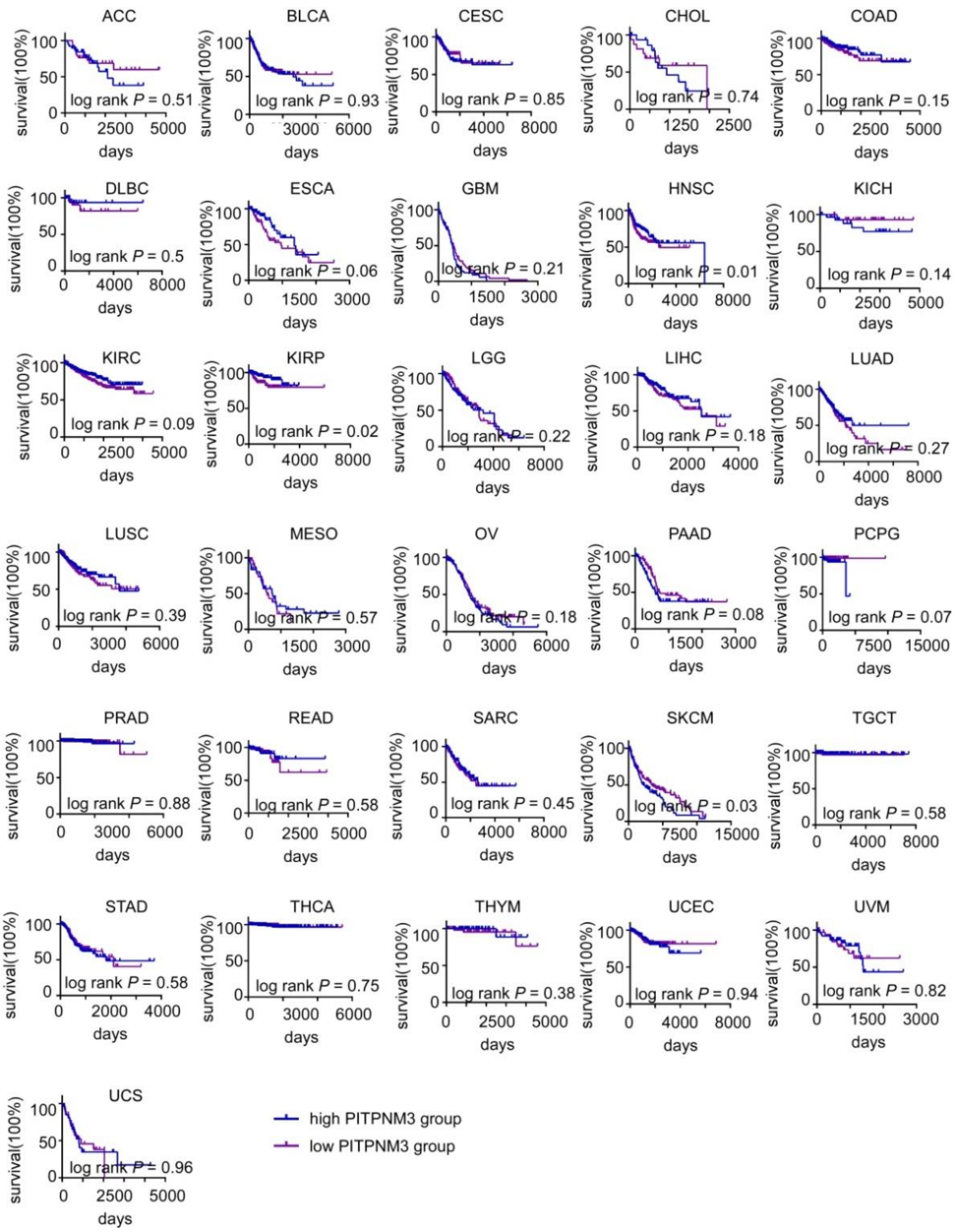

Supplementary Figure S2. Prognostic roles of PITPNM3 in pan-cancer. Pan-cancer abbreviations: adrenocortical cancer (ACC), bladder cancer (BLCA), breast cancer (BRCA), cervical cancer (CESC), cholangiocarcinoma (CHOL), colon cancer (COAD), large B-cell lymphoma (DLBC), esophageal cancer (ESCA), glioblastoma (GBM), head and neck cancer (HNSC), kidney chromophobe cancer (KICH), kidney clear cell carcinoma (KIRC), kidney papillary cell carcinoma (KIRP), acute myeloid leukemia (LAML), lower grade glioma (LGG), liver cancer (LIHC), lung adenocarcinoma (LUAD), lung squamous cell carcinoma (LUSC), mesothelioma (MESO), ovarian cancer (OV), pancreatic cancer (PAAD), pheochromocytoma and paraganglioma (PCPG), prostate cancer (PRAD), rectal cancer (READ), sarcoma (SARC), melanoma (SKCM), stomach cancer (STAD), testicular cancer (TGCT), thyroid cancer (THCA), thymoma (THYM), endometrioid cancer (UCEC), uterine carcinosarcoma (UCS), ocular melanomas (UVM)

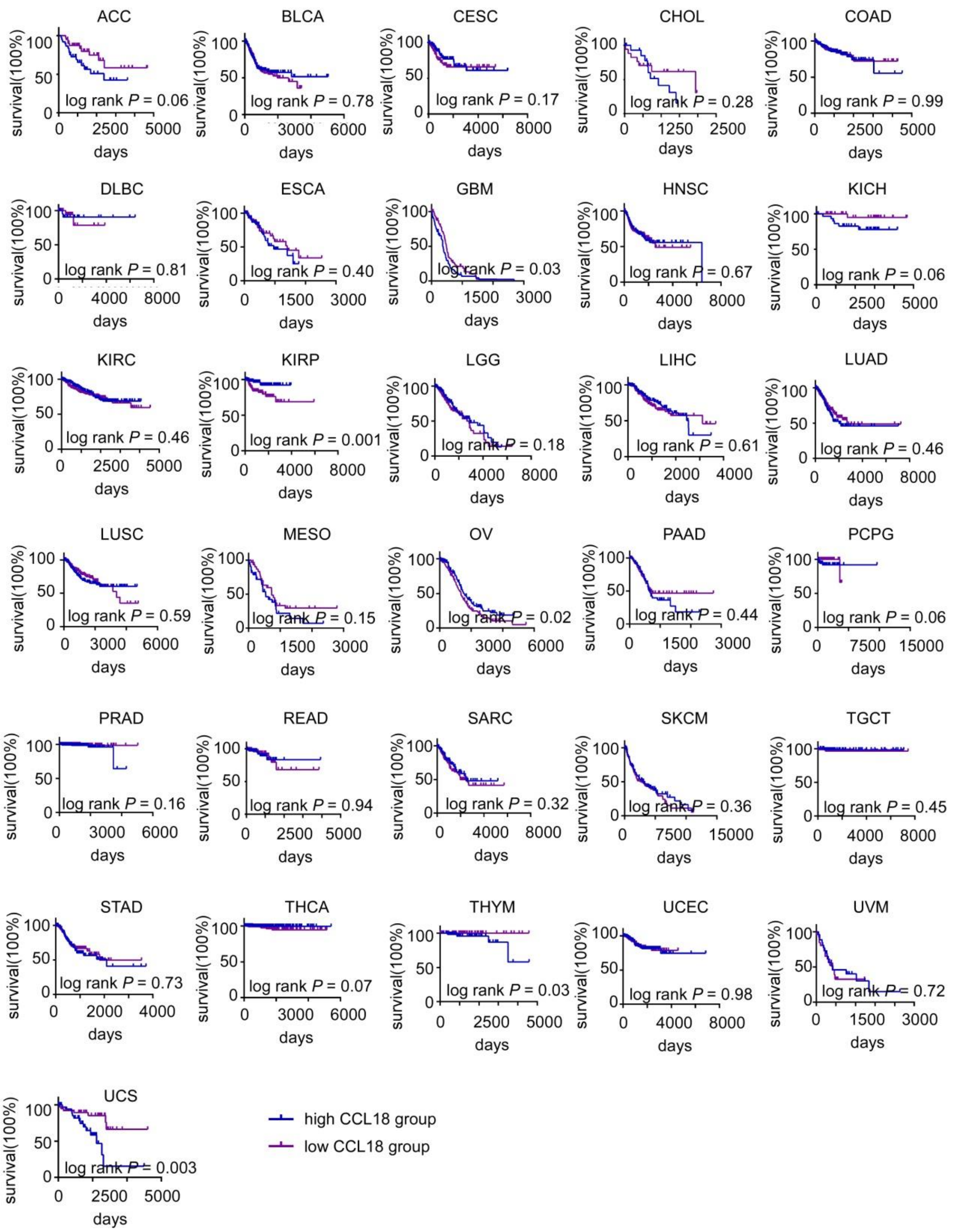

Supplementary Figure S3. Prognostic roles of CCL18 in pan-cancer of TCGA datasets.

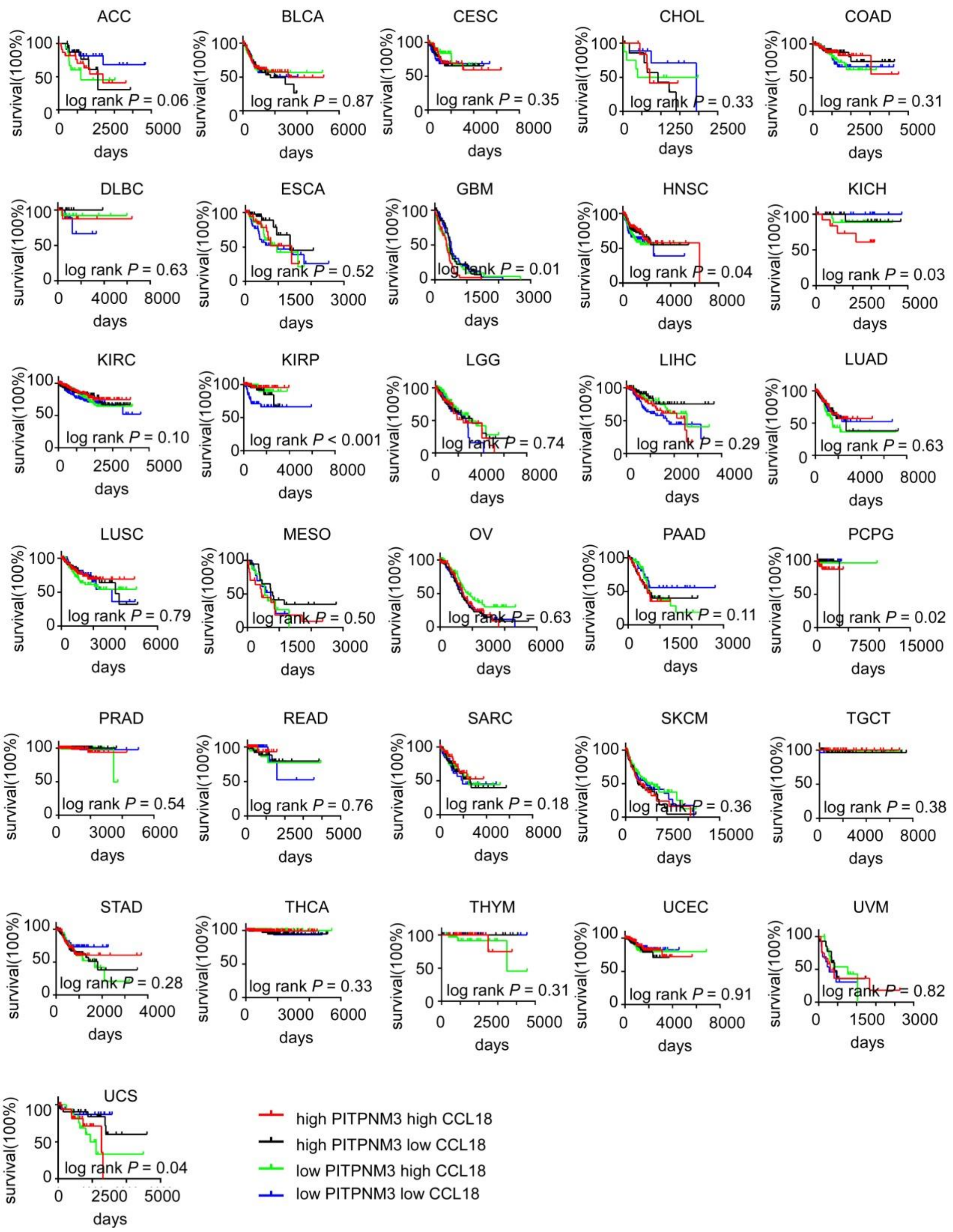

Supplementary Figure S4. Prognostic roles of co-expression of CCL18 and PITPNM3 in pan-cancer of TCGA datasets. The log rank *P* value is compared between PITPNM3^{high} CCL18^{high} and PITPNM3 ^{low} CCL18^{low}.

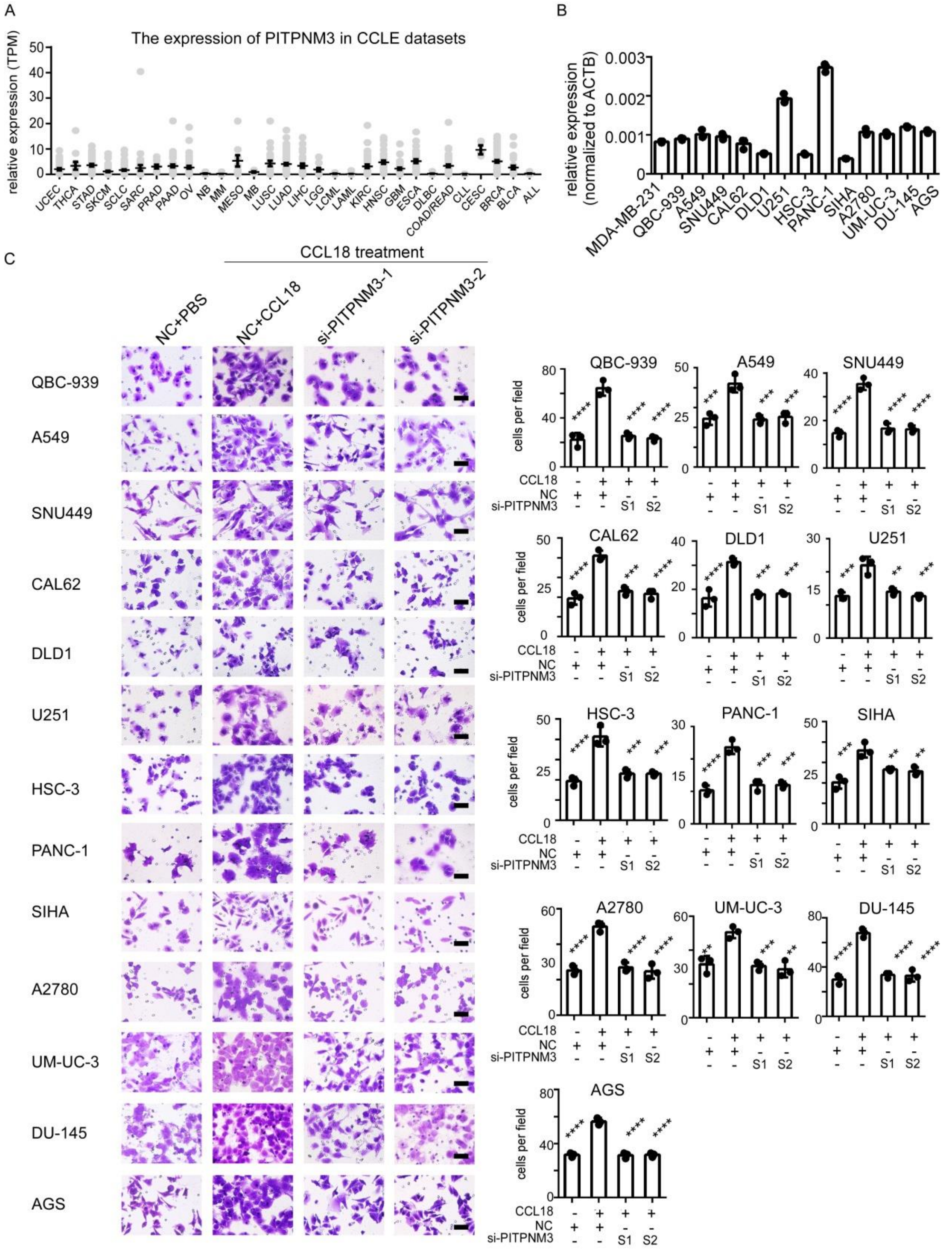

Supplementary Figure S5. The functional roles of CCL18-PITPNM3 signaling pathways in pan-cancer. A. The relative expression of PITPNM3 in CCLE datasets. B. The relative expression of PITPNM3 in 13 different types of cancer cell lines which was detected by qPCR. C. Representing images of cancer metastasis promotion effects of PITPNM3 in 13 different types of cancer cell lines. All data were expressed as means with±SD of three independent experiments and data was compared with the CCL18 treated group. Scale bar 30μm.* *P*< 0.05, ** *P*< 0.01, *** *P*< 0.001, **** *P*< 0.0001

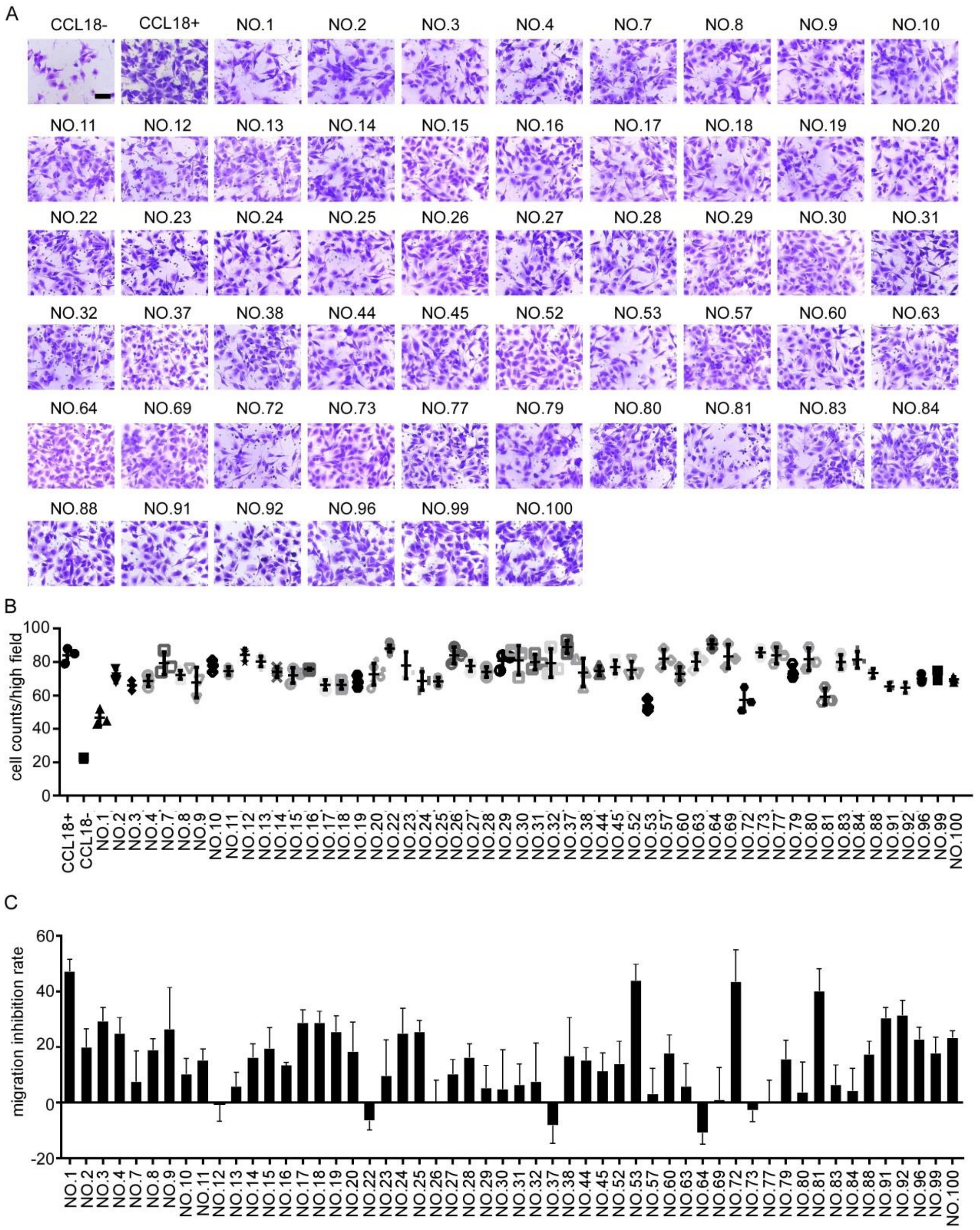

Supplementary Figure S6. Screening of anti-PITPNM3 compounds by transwell migration assay among 54 low toxicity small molecular compounds. A. Representing images of anti-migration effects of anti-PITPNM3 compounds on CCL18-PITPNM3 promoted migration in MDA-MB-231. B. Three independent experiments of migration assay in MDA-MB-231. C. Migration inhibition rates of 54 anti-PITPNM3 compounds. Scale bar 30μm.

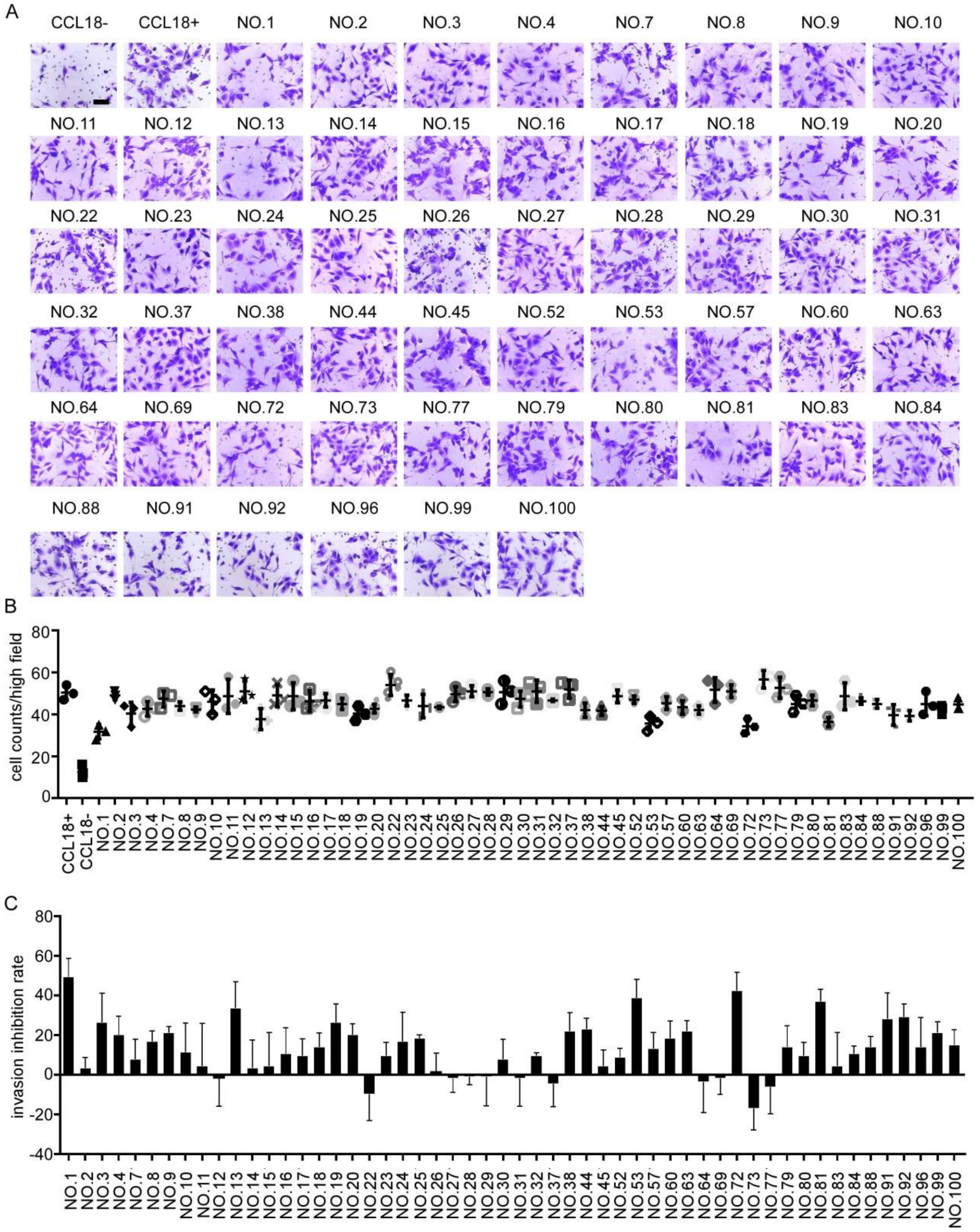

Supplementary Figure S7. Screening of anti-PITPNM3 compounds by transwell invasion assay among 54 low toxicity small molecular compounds. A. Representing images of anti-invasion effects of anti-PITPNM3 compounds on CCL18-PITPNM3 promoted invasion in MDA-MB-231. B. Three independent experiments of invasion assay in MDA-MB-231. C. Invasion inhibition rates of 54 anti-PITPNM3 compounds. Scale bar 30μm.

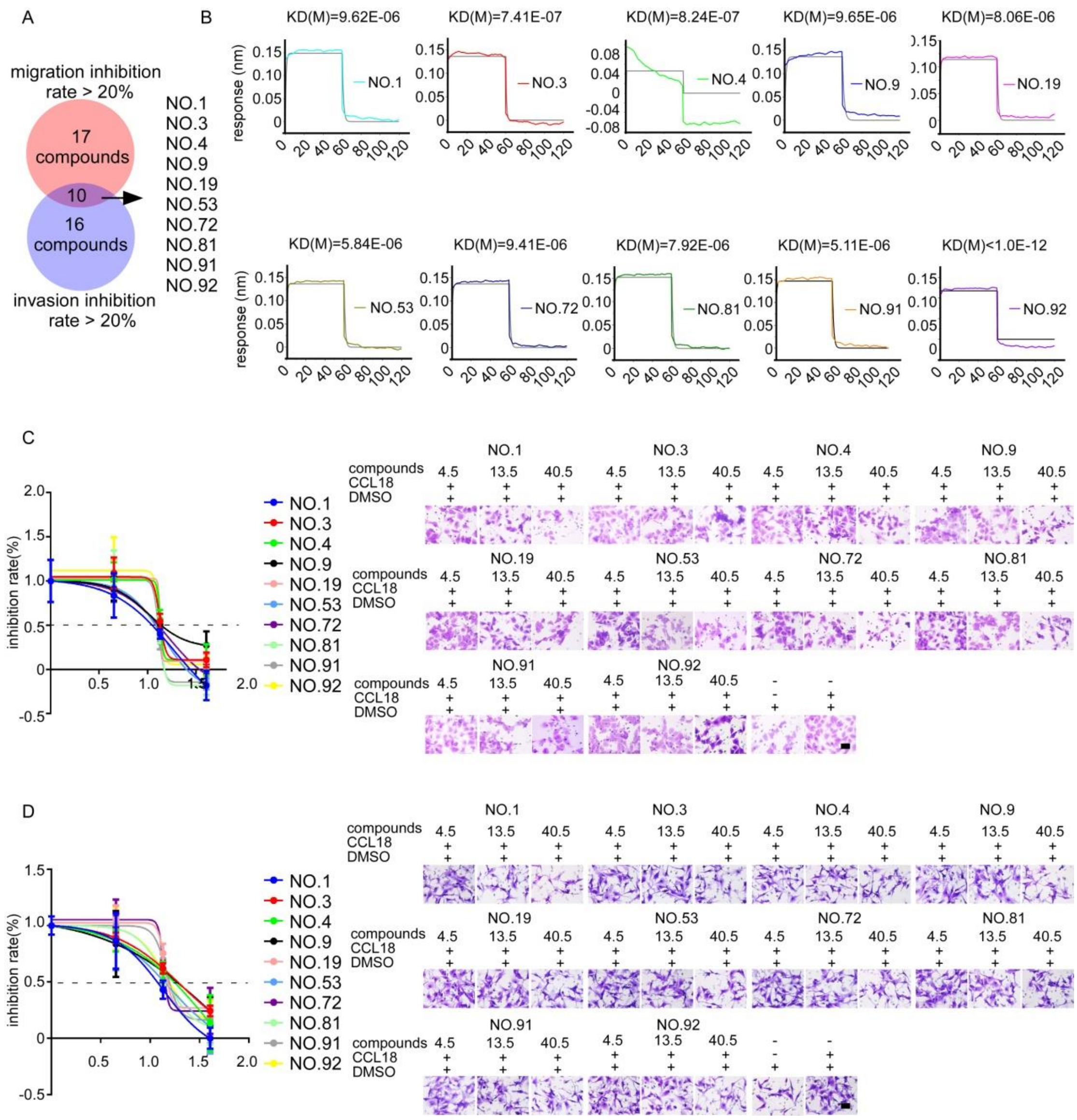

Supplementary Figure S8. Screening of anti-PITPNM3 compounds by Forterbio binding affinity assay and transwell assay among 10 small molecular compounds. A. Intersection of anti-PITPNM3 compounds with their invasion inhibition rates over 20% and migration inhibition rates over 20%. B. Fitting curve of Forterbio binding response of anti-PITPNM3 compounds. C. Representing images of anti-migration and anti-invasion effects of 10 small molecular compounds. The EC50 fitting curve of the 10 small molecular compounds. All data were expressed as means with±SD of three independent experiments. Scale bar 30μm.

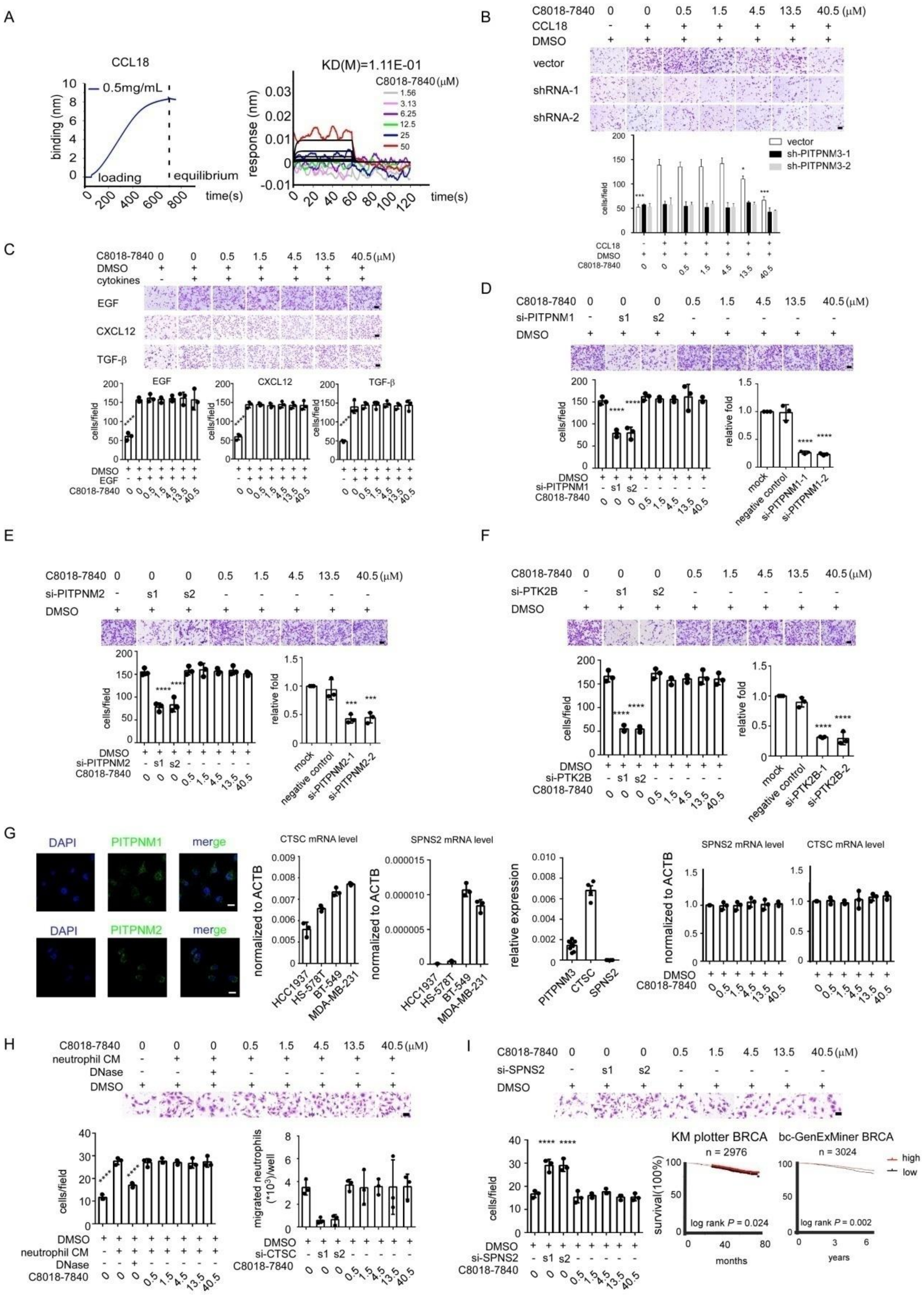

Supplementary Figure S9. The off-target of effects of C8018-7840. A. Binding affinity between human recombinant CCL18 and C8018-7840. B. Transwell assay between vector MDA-MB-231 cells and sh-PITPNM3 MDA-MB-231 cells. Scale bar 100μm. C. C8018-7840 barely inhibits EGF, TGF-β and CXCL12 mediated migration in MDA-MB-231 cells. Scale bar 100μm. D. C8018-7840 barely inhibits MDA-MB-231 migration under normal condition, while knockdown of PITPNM1 significantly abrogates cell migration.

Small interference RNAs targeting PITPNM1 can significantly inhibit PITPNM1 mRNA level in MDA-MB-231. Scale bar 100μm. E. C8018-7840 barely inhibits MDA-MB-231 migration under normal condition, while knockdown of PITPNM2 significantly abrogates cell migration. Small interference RNAs targeting PITPNM2 can significantly inhibit PITPNM1 mRNA level in MDA-MB-231. Scale bar 100μm. F. C8018-7840 barely inhibits MDA-MB-231 migration while knockdown of PTK2B significantly abrogates cell migration. Scale bar 100μm. G. PITPNM1 and PITPNM2 locates in the cytoplasm. The relative expression of CTSC and SPNS2 in breast cancer cell lines. Treatment of C8018-7840 barely affect the expression of CTSC and SPNS2 which is confirmed by qPCR. H. The effects of C8018-7840 on neutrophils conducted breast cancer cell migration. Scale bar 30μm. The effects of C8018-7840 on CTSC conducted chemotaxis of neutrophils. I. C8018-7840 barely inhibits MDA-MB-231 migration while knockdown of SPNS2 significantly abrogates cell migration. Scale bar 30μm. The prognostic role of SPNS2 in breast cancer in KM plotter datasets and bc-GeneMiner datasets. All data was expressed as means with±SD of three independent experiments. n.s. not significant, * $P < 0.05$, ** $P < 0.01$, *** $P < 0.001$, **** $P < 0.0001$

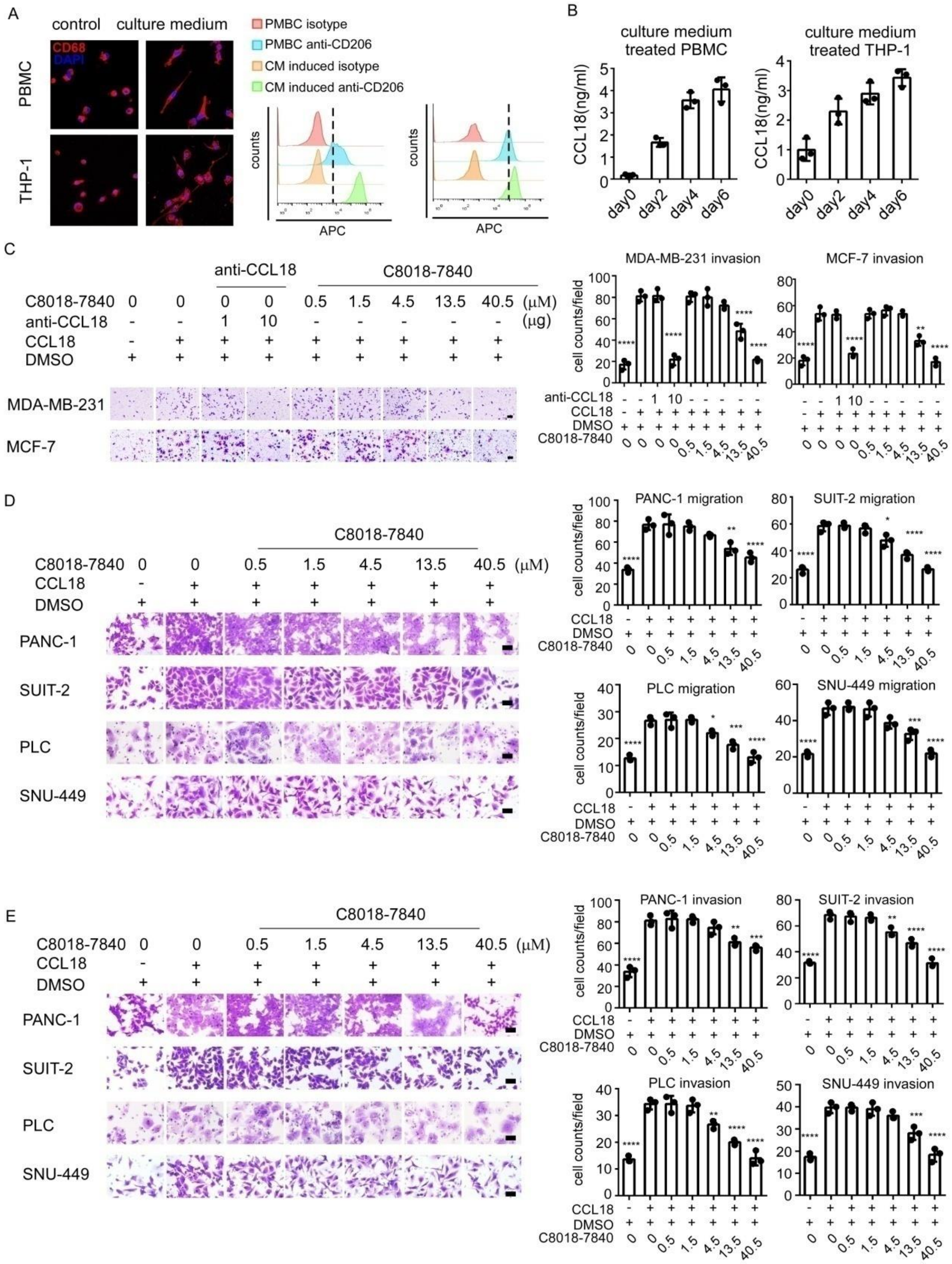

Supplementary Figure S10. The effects of C8018-7840 on the THP-1 macrophages induced metastasis in breast cancer cells. A. Fluorescent CD68/DAPI staining in PBMC and THP-1 induced tumor associated macrophages. CD206 isotype of PBMC and THP-1 induced tumor associated macrophages. B. The protein level of CCL18 in the media of PBMC and THP-1 induced tumor associated macrophages which was confirmed by ELISA. C. The representing images of anti-invasion effects of C8018-7840 on THP1 macrophages-induced invasion in MDA-MB-231 and MCF-7 cells. Scale bar 100μm. D. Transwell migration assay validation of anti-migration effects of C8018-7840 on CCL18 induced invasion in hepatocellular

carcinama cell lines (SUN-449 and PLC) and pancreatic ductal carcinoma (PANC-1 and SUIT-2). Scale bar 30μm. E. Transwell invasion assay validation of anti-invasion effects of C8018-7840 on CCL18 induced invasion in hepatocellular carcinama cell lines (SNU-449 and PLC) and pancreatic ductal carcinoma (PANC-1 and SUIT-2). Scale bar 30μm. All data was expressed as means with±SD of three independent experiments. n.s. not significant, * $P < 0.05$, ** $P < 0.01$, *** $P < 0.001$, **** $P < 0.0001$

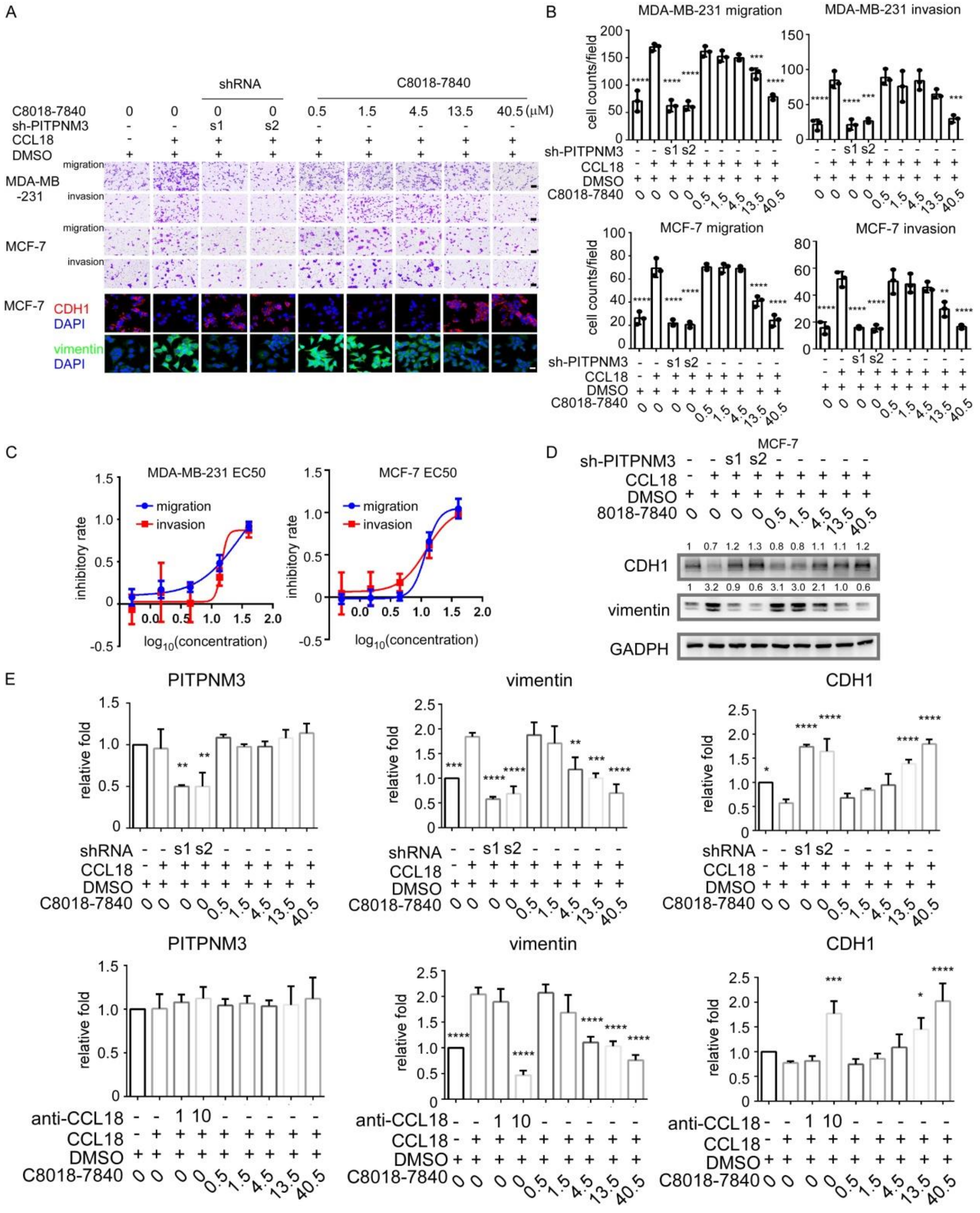

Supplementary Figure S11. The effects of C8018-7840 on PITPNM3 conducted metastasis in breast cancer cells. A. Representing images of anti-migration effects and anti-invasion effects of C8018-7840 on PITPNM3 conducted migration and invasion in MDA-MB-231 and MCF-7 cells. Cells were treated with human recombinant CCL18. Scale bar 100μm. Representing images of anti-EMT effects of C8018-7840 Scale bar 20μm. B. Statistical diagrams of anti-migration effects and anti-invasion effects of C8018-7840 on PITPNM3 conducted migration and invasion in MDA-MB-231 and MCF-7 cells. C. EC50 regression fitting curve of anti-migration and anti-invasion of C8018-7840. D. Inhibition effects of C8018-7840 on EMT in MCF-7 cells which are validated by Western Blot. E. Inhibition effects of C8018-7840 on EMT markers validated by qPCR. All data was expressed as means

with±SD of three independent experiments. All data were expressed as means with±SD of three independent experiments and data was compared with the CCL18 treated group.* P< 0.05, ** P< 0.01, *** P< 0.001, **** P< 0.0001

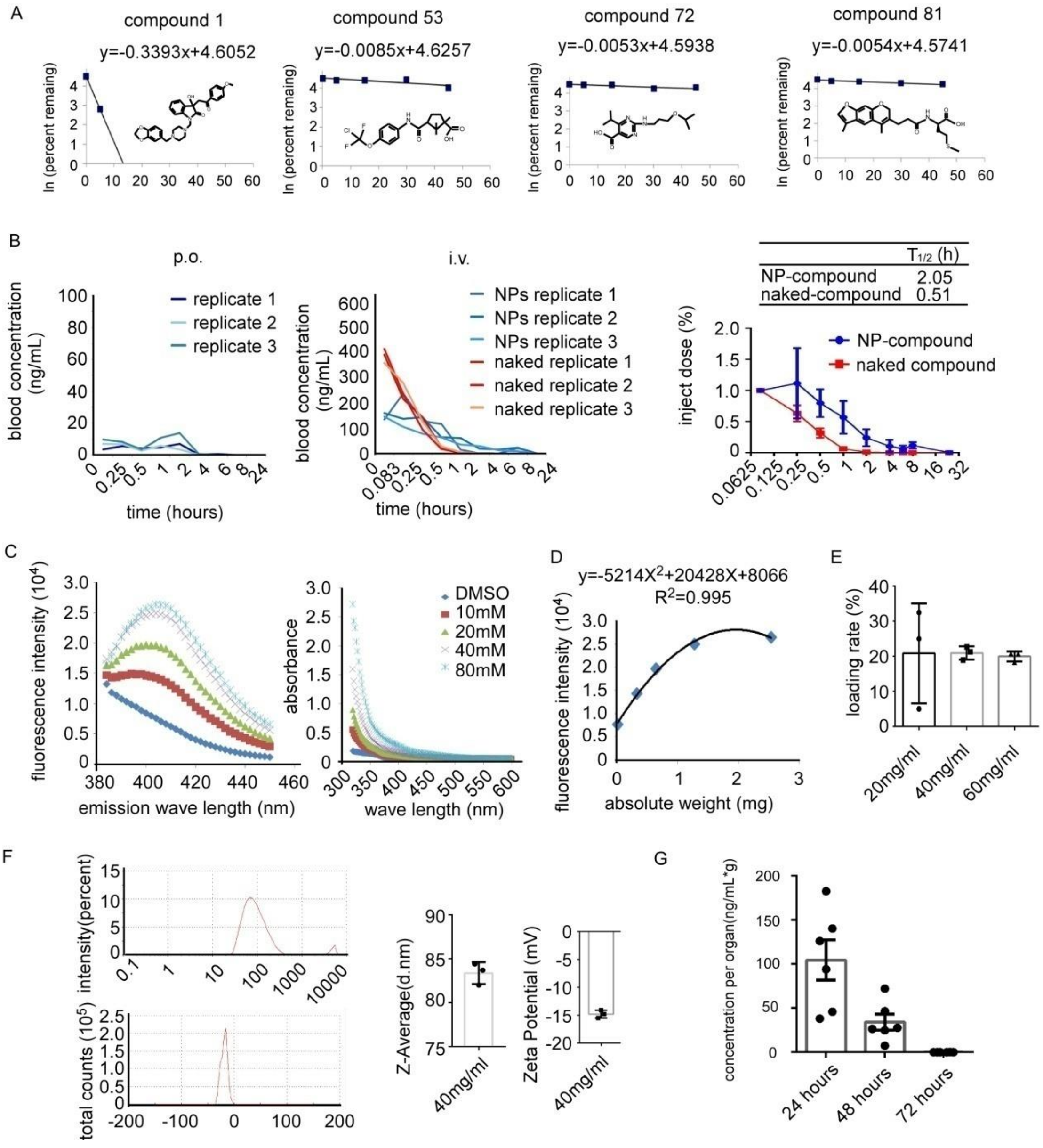

Supplementary Figure S12. Characteristics of C8018-7840 loaded PEG-PLGA NPs. A. Anti-PITPNM3 compounds metabolized by rat liver microsomes. B. The pharmacokinetics of C8018-7840 and NP-C8018-7840 in rats which were dosed of p.o. or i.v. administration. C. The excitation-emission and absorbance scan of C8018-7840. C8018-7840 exhibited a specific fluorescence intensity at 390~410nm excitation wave length D. The regression of C8018-7840 weight and fluorescence intensity regression equation with $R^2 > 0.99$. E. Loading rates of NP-C8018-7840. F. Average size and zeta potential of NP- C8018-7840. D. The accumulation of NP-C8018-7840 in tumor tissue which was detected by LC-MS/MS after 24, 48 and 72 hours tail vein injection.

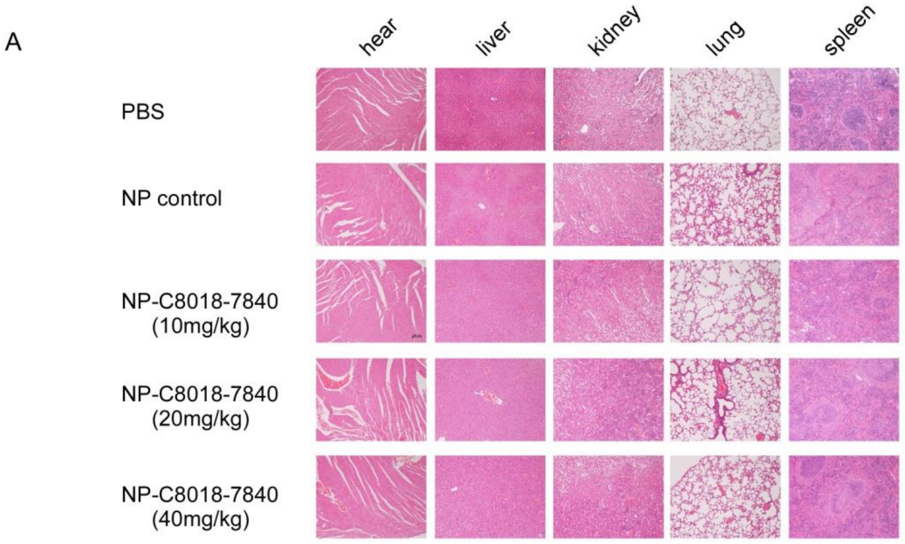

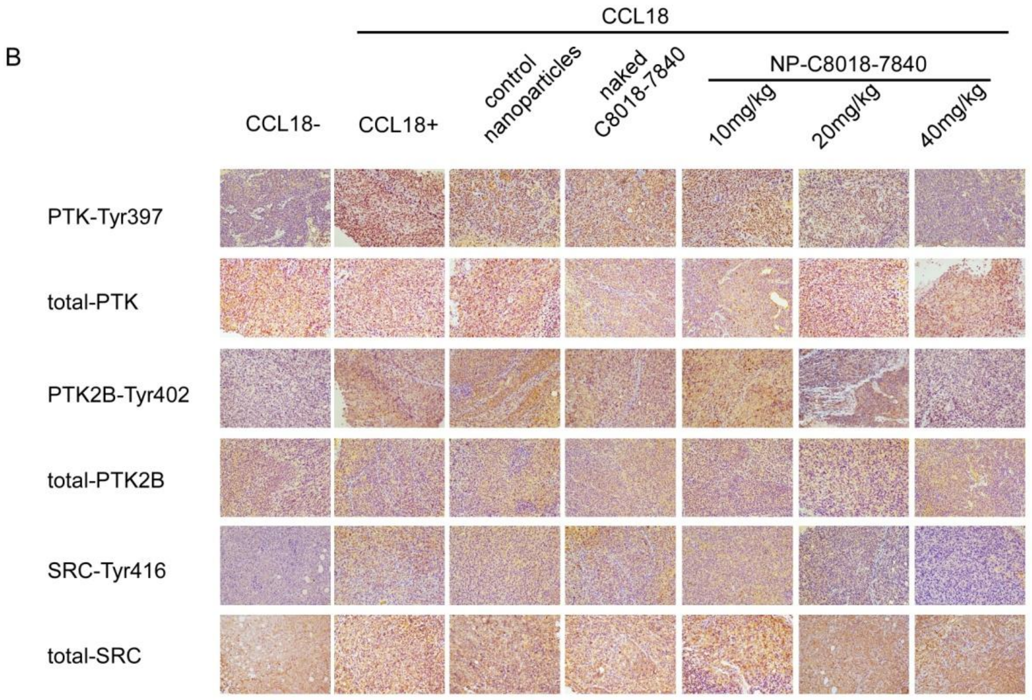

Supplementary Figure S13. The efficacy of NP-C8018-7840 *in vivo*. A. H&E of different organs of BALB/c nude mice treated with PBS, control NPs and NP-C8018-7840. Scale bar: 100μm. B. IHC staining of phosphorylation PTK2B at Tyr402, PTK at Tyr397,SRC at Tyr416, total-PTK2B, total-PTK, and total-SRC of primary tumor harvested from mice bearing MDA-MB-231-Luc orthotopic tumor.

Supplementary Table S1. Docking performance of anti-PITPNM3 compounds

ID	ID number	fmla structure	Molweight	Docking performance
NO.1	8018-7840	C30H31N3O6	529.6	-12.51
NO.2	D340-1837	C24H25ClN4O4	468.94	-11.91
NO.3	6528-0117	C28H28ClN3O5	522.01	-11.67
NO.4	S567-0119	C24H34N4O3	426.56	-11.63
NO.5	0449-0098	C23H34O6	406.52	-11.56
NO.6	Y040-8554	C29H32BrNO5	554.49	-11.52
NO.7	3141-0169	C25H19NO6	429.43	-11.5
NO.8	6528-0237	C28H29N3O5	487.56	-11.33
NO.9	2372-3741	C20H12N2O6S	408.39	-10.98
NO.10	N039-0046	C30H50O5	490.73	-10.98
NO.11	S567-0086	C24H36N4O3	428.58	-10.88
NO.12	N064-0038	C30H47NO3	469.71	-10.72
NO.13	C650-0091	C23H19ClN4O3S	466.95	-10.59
NO.14	K292-1858	C18H13BrN4O4S2	493.36	-10.48
NO.15	G857-1906	C21H22N4O4S	426.5	-10.44
NO.16	J031-2277	C17H19N3O4S	361.42	-10.26
NO.17	7287-0201	C18H17BrN2O5S	453.31	-10.22
NO.18	K292-1780	C23H21BrN4O4S2	561.48	-10.19
NO.19	S567-0112	C18H32N4O3S	384.54	-10.01
NO.20	N040-0002	C44H70O15	839.04	-10
NO.21	N064-0033	C31H50O3	470.74	-9.99
NO.22	H025-0218	C20H18F2N2O4S	420.44	-9.98
NO.23	8640-0345	C16H21N3O3S2	367.49	-9.79
NO.24	S567-0117	C19H28N4O3S2	424.59	-9.72
NO.25	0449-0076	C23H31NO5	401.51	-9.68
NO.26	8018-4543	C24H25FN2O5	440.48	-9.66
NO.27	D030-1011	C17H25ClN2O3S	372.92	-9.64
NO.28	Y200-5859	C15H20N2O6S	356.4	-9.62
NO.29	Y020-9283	C20H17FN4O4	396.38	-9.61
NO.30	S596-0660	C22H36N4O4	420.56	-9.58
NO.31	J031-1753	C16H17N3O4S	347.4	-9.53
NO.32	8012-6937	C23H29F3N2O4	454.49	-9.51
NO.33	Y200-4670	C15H18N4O5	334.33	-9.5
NO.34	8015-3420	C21H26N2O6S	434.52	-9.49
NO.35	4896-4130	C21H19BrN4O5S	519.38	-9.46
NO.36	4358-1606	C21H13Cl2N3O2	446.72	-9.41
NO.37	V030-2304	C25H24N2O6S	480.54	-9.41
NO.38	4900-0021	C26H30N2O	386.54	-9.38
NO.39	D086-0259	C18H13N3O3S	351.39	-9.37
NO.40	4676-0660	C20H19N3O5S	413.46	-9.36
NO.41	C109-0679	C23H17BrClN3O5	530.77	-9.33

NO.42	D030-0737	C17H26N2O3S	338.47	-9.28
NO.43	8012-7758	C25H22N2O5S	462.53	-9.27
NO.44	8640-0003	C16H16N4O4S	360.39	-9.25
NO.45	8640-0002	C17H18N4O4S	374.42	-9.22
NO.46	N026-0105	C30H44O4	468.68	-9.16
NO.47	8179-0053	C26H24N2O4	428.49	-9.12
NO.48	G857-1929	C20H18Cl2N4O3S	465.36	-9.06
NO.49	D034-0029	C23H22N2O3	374.44	-9.05
NO.50	4644-0543	C20H24ClNO6S	441.93	-9.01
NO.51	V001-3187	C20H28ClNO6	413.9	-9
NO.52	S629-0005	C13H17NO5S	299.35	-8.98
NO.53	8011-9111	C17H20ClF2NO4	375.8	-8.96
NO.54	0407-0023	C23H34O5	390.52	-8.93
NO.55	8179-0043	C25H22N2O4	414.47	-8.91
NO.56	C798-0513	C26H34N2O6	470.57	-8.82
NO.57	S567-0097	C20H33N3O3	363.5	-8.82
NO.58	Y040-2697	C24H29NO5	411.5	-8.82
NO.59	N088-0024	C20H36O5	356.51	-8.81
NO.60	S596-0516	C20H33N3O3	363.5	-8.79
NO.61	5915-0569	C11H11N3O5S2	329.36	-8.78
NO.62	D005-0089	C20H15FN2O3S	382.42	-8.76
NO.63	Y040-1946	C21H14O5	346.34	-8.73
NO.64	Y200-4658	C14H16N4O5	320.31	-8.71
NO.65	8539-0478	C13H13NO5	263.25	-8.69
NO.66	D340-2271	C16H26N4O3	322.41	-8.68
NO.67	8019-8134	C22H16FNO5S	425.44	-8.66
NO.68	Y041-1956	C19H23NO6S	393.46	-8.62
NO.69	0449-0090	C22H30O6	390.48	-8.5
NO.70	8640-0197	C21H27N3O6S	449.53	-8.46
NO.71	S511-0685	C15H26N2O4	298.39	-8.46
NO.72	S272-2144	C14H23N3O3	281.36	-8.43
NO.73	S596-1146	C18H31N3O3	337.47	-8.41
NO.74	7202-4704	C15H14N2O5S	334.35	-8.36
NO.75	7407-0184	C18H14BrNO5	404.22	-8.33
NO.76	G856-1330	C19H12F3N3O4S	435.38	-8.22
NO.77	S567-0100	C19H29FN2O3S	384.52	-8.21
NO.78	C660-1095	C12H11FN2O5S	314.29	-8.18
NO.79	Y200-5933	C18H24N2O4S	364.47	-8.17
NO.80	Y041-4804	C15H17N3O4S	335.38	-8.16
NO.81	Y041-2226	C21H23NO6S	417.48	-8.04
NO.82	3448-6882	C14H17NO4	263.3	-8.03
NO.83	Y200-5898	C17H22N2O4S	350.44	-8.01
NO.84	G208-0184	C24H25N3O4	419.48	-8
NO.85	N010-0009	C20H34O4	338.49	-7.97

NO.86	8415-1667	C10H9N3O3S	251.27	-7.93
NO.87	C660-1680	C14H18N4O3	290.32	-7.91
NO.88	4393-0234	C19H24O5	332.4	-7.83
NO.89	M506-0385	C22H15FN2O2	358.38	-7.68
NO.90	5610-0065	C24H32O5	400.52	-7.66
NO.91	K906-0466	C10H9NO6S	271.25	-7.61
NO.92	8012-4528	C18H28N4O4	364.45	-7.59
NO.93	N017-0019	C26H42O3	402.62	-7.57
NO.94	6721-1476	C15H10ClNO5S2	383.83	-7.55
NO.95	8004-6664	C22H34O3	346.51	-7.51
NO.96	5555-0349	C16H23NO4	293.37	-7.5
NO.97	6077-0301	C13H12FNO4S	297.31	-7.41
NO.98	C200-7498	C19H11NO4S	349.37	-7.35
NO.99	1781-2035	C17H19NO6	333.34	-7.33
NO.100	8015-0046	C16H21N3O4	319.36	-7.29

Supplementary Table S2. Toxicity of anti-PITPNM3 compounds

Toxicity of top 100 compounds with highest *in silico* scores in MDA-MB-231 cells

ID	Inhibition rate (%)	ID	Inhibition rate (%)	ID	Inhibition rate (%)	ID	Inhibition rate (%)
NO.1	1.81	NO.2	8.06	NO.3	4.60	NO.4	1.41
NO.5	39.30	NO.6	13.59	NO.7	3.57	NO.8	5.14
NO.9	0.65	NO.10	3.51	NO.11	0.71	NO.12	0.42
NO.13	1.38	NO.14	2.48	NO.15	0.47	NO.16	2.32
NO.17	0.20	NO.18	4.31	NO.19	2.15	NO.20	4.31
NO.21	24.33	NO.22	1.86	NO.23	2.43	NO.24	4.08
NO.25	1.78	NO.26	2.32	NO.27	1.13	NO.28	2.17
NO.29	2.04	NO.30	0.12	NO.31	1.22	NO.32	3.11
NO.33	14.32	NO.34	18.36	NO.35	17.66	NO.36	15.56
NO.37	6.08	NO.38	8.23	NO.39	11.21	NO.40	10.96
NO.41	16.04	NO.42	15.28	NO.43	21.03	NO.44	10.00
NO.45	6.39	NO.46	14.97	NO.47	18.21	NO.48	13.32
NO.49	13.96	NO.50	15.15	NO.51	13.80	NO.52	7.41
NO.53	16.48	NO.54	43.02	NO.55	16.32	NO.56	11.39
NO.57	8.48	NO.58	18.54	NO.59	12.64	NO.60	2.63
NO.61	17.56	NO.62	40.09	NO.63	1.65	NO.64	2.61
NO.65	11.52	NO.66	12.17	NO.67	14.91	NO.68	12.79
NO.69	6.22	NO.70	11.92	NO.71	11.33	NO.72	7.85
NO.73	8.07	NO.74	11.93	NO.75	15.47	NO.76	14.65
NO.77	9.31	NO.78	14.65	NO.79	7.75	NO.80	7.56
NO.81	9.00	NO.82	10.16	NO.83	8.52	NO.84	0.97
NO.85	14.64	NO.86	10.40	NO.87	12.49	NO.88	6.13
NO.89	12.20	NO.90	15.42	NO.91	7.43	NO.92	3.15
NO.93	18.53	NO.94	20.14	NO.95	14.78	NO.96	6.66
NO.97	13.60	NO.98	16.03	NO.99	6.93	NO.100	0.41

Supplementary Table S3. Inhibition rate of anti-PITPNM3 compounds of CCL18-PITPNM3 induced migration.

ID	Inhibition rate (%)	ID	Inhibition rate (%)	ID	Inhibition rate (%)	ID	Inhibition rate (%)
NO.1	47.28	NO.2	20.10	NO.3	29.34	NO.4	25.00
NO.7	7.60	NO.8	19.02	NO.9	26.63	NO.10	10.32
NO.11	15.21	NO.12	-0.54	NO.13	5.97	NO.14	16.30
NO.15	19.56	NO.16	13.58	NO.17	28.80	NO.18	28.80
NO.19	25.54	NO.20	18.47	NO.22	-6.52	NO.23	9.78
NO.24	25.00	NO.25	25.54	NO.26	0.00	NO.27	10.32
NO.28	16.30	NO.29	5.43	NO.30	4.89	NO.31	6.52
NO.32	7.60	NO.37	-8.15	NO.38	16.84	NO.44	15.21
NO.45	11.41	NO.52	14.13	NO.53	44.02	NO.57	3.26
NO.60	17.93	NO.63	5.97	NO.64	-10.86	NO.69	1.08
NO.72	43.47	NO.73	-2.71	NO.77	0.00	NO.79	15.76
NO.80	3.80	NO.81	40.21	NO.83	6.52	NO.84	4.34
NO.88	17.39	NO.91	30.43	NO.92	31.52	NO.96	22.82
NO.99	17.93	NO.100	23.36				

Supplementary Table S4. Inhibition rate of anti-PITPNM3 compounds of CCL18-PITPNM3 induced invasion.

ID	Inhibition rate (%)	ID	Inhibition rate (%)	ID	Inhibition rate (%)	ID	Inhibition rate (%)
NO.1	49.56	NO.2	3.54	NO.3	26.55	NO.4	20.35
NO.7	7.96	NO.8	16.81	NO.9	21.24	NO.10	11.50
NO.11	4.42	NO.12	-1.77	NO.13	33.63	NO.14	3.54
NO.15	4.42	NO.16	10.62	NO.17	9.73	NO.18	14.16
NO.19	26.55	NO.20	20.35	NO.22	-9.73	NO.23	9.73
NO.24	16.81	NO.25	18.58	NO.26	1.77	NO.27	-1.77
NO.28	-0.88	NO.29	-0.88	NO.30	7.96	NO.31	-1.77
NO.32	9.73	NO.37	-4.42	NO.38	22.12	NO.44	23.01
NO.45	4.42	NO.52	8.85	NO.53	38.94	NO.57	13.27
NO.60	18.58	NO.63	22.12	NO.64	-3.54	NO.69	-1.77
NO.72	42.48	NO.73	-16.81	NO.77	-6.19	NO.79	14.16
NO.80	9.73	NO.81	37.17	NO.83	4.42	NO.84	10.62
NO.88	14.16	NO.91	28.32	NO.92	29.20	NO.96	14.16
NO.99	21.24	NO.100	15.04				
